# Prolonged Infections and Inflammatory Diseases in Common Variable Immune Deficiency as a Cause of AA Amyloidosis

**DOI:** 10.3390/jcm15114030

**Published:** 2026-05-22

**Authors:** Elena V. Reznik, Maksim D. Iarovoi, Tatiana S. Romanova, Elena A. Latysheva, Tatiana V. Latysheva, Nikolay A. Nazarov, Anastasiia A. Buianova, Iuliia A. Vasiliadis, Zhanna A. Repinskaia, Vladislav A. Strutynsky, Georgy N. Golukhov

**Affiliations:** 1 Pirogov Russian National Research Medical University, 1, Ostrovityanova St., 117997 Moscow, Russia; reznik_ev@rsmu.ru (E.V.R.); ea.latysheva@nrcii.ru (E.A.L.); anastasiiabuianova97@gmail.com (A.A.B.); julia.vasiliadis@gmail.com (I.A.V.); repinskaia@gmail.com (Z.A.R.); gkb31@zdrav.mos.ru (G.N.G.); 2 Clinical Hospital № 31 Named After G.M. Savelieva of the Moscow Health Department, 42, Bldg. 4, Lobachevsky St., 119415 Moscow, Russia; ts_romanova@mail.ru (T.S.R.); tv.latysheva@nrcii.ru (T.V.L.); 3 NRC Institute of Immunology FMBA of Russia, 24, Kashirskoe Highway, 115522 Moscow, Russia; 5898050@gmail.com; 4 Institute of Nuclear Medicine, Moscow Region, Klyazma Quarter, Bldg. 300, 141432 Khimki, Russia; magama91@gmail.com

**Keywords:** amyloidosis, AA amyloidosis, inborn error of immunity, immunodeficiency, common variable immune deficiency

## Abstract

**Background/Objectives**: AA amyloidosis is a serious complication of chronic inflammation, which may arise in the setting of inborn errors of immunity (IEIs) due to recurrent or persistent infections. Common variable immunodeficiency (CVID) is the most frequent symptomatic IEI in adults, yet its association with secondary AA amyloidosis remains rarely reported. **Case presentation**: We describe a 37-year-old male with a history of recurrent pneumonia, chronic sinusitis, and osteomyelitis with sepsis since childhood. At age 33, he developed bilateral pneumonia after COVID-19, followed by repeated lower respiratory tract infections. At age 36, nephrotic syndrome (proteinuria 10.69 g/day, hypoalbuminemia) led to kidney and gastric mucosa biopsies, which confirmed AA amyloidosis. Immunological workup revealed panhypogammaglobulinemia (IgG 0.1 g/L, IgA 0.01 g/L, IgM 0.28 g/L), markedly reduced switched memory B cells, and an inverted CD4^+^/CD8^+^ ratio. Chest CT showed bronchiectasis, bronchiolitis, and mediastinal lymphadenopathy. Whole-exome sequencing excluded known monogenic IEIs, autoinflammatory, or hereditary amyloidosis genes; a heterozygous likely pathogenic variant in *ODAD2* (associated with primary ciliary dyskinesia) was considered incidental. A diagnosis of CVID with secondary AA amyloidosis was established. **Conclusions**: This case illustrates that CVID may remain undiagnosed for decades and present with secondary AA amyloidosis as the first major complication. In any patient with nephrotic syndrome and a history of recurrent or unusual infections, an IEI should be actively excluded. Early recognition of CVID and appropriate immunoglobulin replacement therapy can prevent infectious exacerbations and potentially halt amyloid progression.

## 1. Introduction

Amyloidosis is a group of diseases characterized by the deposition of fibrillar glycoprotein amyloid in body tissues. This protein exhibits birefringence—appearing as apple-green under polarized light in Congo red-stained preparations [1].

Amyloid infiltration leads to organomegaly, cellular death, and functional impairment. The clinical presentation is highly variable and may include amyloid nephropathy, heart failure, autonomic nervous system dysfunction, hepatocellular failure, gastrointestinal dyspepsia, and ocular and cutaneous involvement, among others [1,2].

Amyloidosis should be considered in the differential diagnosis when one or more of the following conditions are present: nephrotic syndrome of non-diabetic origin, restrictive/hypertrophic cardiomyopathy, chronic heart failure, hepatosplenomegaly, carpal tunnel syndrome, unexplained facial or cervical purpura, and macroglossia [1].

To date, approximately 36 precursor proteins leading to 36 different types of amyloidosis have been identified [3].

The most common types of amyloidosis are AA, AL, and ATTR amyloidosis, while rarer forms include Aβ2M, AGel, AApoAI, AFib, Aβ2, APrPScr, AANF, AIAPP, ACal, and ACys amyloidosis [4].

AA amyloidosis is a secondary form of amyloidosis that develops in the context of chronic inflammatory diseases, though idiopathic AA amyloidosis has also been described. The pathogenesis involves elevated serum concentrations of serum amyloid A (SAA), a protein structurally related to C-reactive protein (CRP), which rises in response to inflammation [5].

Conditions associated with AA amyloidosis include chronic infectious diseases (tuberculosis, osteomyelitis, bronchiectasis, aspergillosis, cystic fibrosis, etc.), rheumatological disorders (rheumatoid arthritis, psoriatic arthritis, spondyloarthritis, gout, Takayasu arteritis, Behçet’s disease), autoinflammatory syndromes (familial Mediterranean fever, cryopyrin-associated periodic syndrome, tumor necrosis factor receptor-associated periodic syndrome (TRAPS), hyperimmunoglobulinemia D with periodic fever syndrome, among others), as well as malignant neoplasms (Hodgkin’s lymphoma, non-Hodgkin’s lymphoma, Waldenström’s macroglobulinemia, and solid tumors of various localizations) [6].

Diagnosis of AA amyloidosis is established by biopsy with Congo red staining and polarized light microscopy, followed by amyloid typing. Serological methods are also recommended to identify SAA, κ and λ immunoglobulin light chains, and transthyretin [1].

Infections are one of the causes of amyloidosis, and an inborn error of immunity (IEI) is one of the causes of chronic infectious diseases. Secondary AA amyloidosis may also develop in the setting of both an IEI and secondary immunodeficiencies (e.g., human immunodeficiency virus, HIV infection). According to Gomzikova et al., secondary amyloidosis of the lungs has been observed in patients with cyclic neutropenia, common variable immunodeficiency (CVID), and hyper-IgM syndrome [7]. Although IEIs are genetically determined, many present years or even decades after birth. The most common IEI in adults is antibody deficiency. These disorders are characterized by a relatively favorable course and a late onset, with two peaks of clinical manifestation: in childhood (2–5 years) and in young adulthood (18–25 years) [7].

CVID is the most frequent IEI associated with antibody deficiency (prevalence: 1:10,000–1:50,000). Historically, CVID was defined as hypogammaglobulinemia affecting ≥2 immunoglobulin classes, impaired antibody response to infections and vaccination, and clinical manifestations including infectious and/or non-infectious complications (autoimmunity, granulomatous disease, inflammatory bowel disease-like syndromes, lymphoproliferation, etc.). The laboratory criteria for CVID diagnosis in adults include reduced IgG (<4.5 g/L) and IgA, with or without reduced IgM, along with decreased switched memory B cells (CD19^+^CD27^+^IgD^−^) in the absence of profound T-cell defects. With advances in molecular genetics, monogenic disorders (e.g., cytotoxic T-lymphocyte associated protein 4 deficiency, lipopolysaccharide-responsive and beige-like anchor protein deficiency, activated PI3K delta syndrome, STAT3-GOF) have been distinguished from CVID, yet most patients without identified mutations remain classified under CVID. The pathogenesis is considered to involve an interplay between genetic polymorphisms and environmental factors. The clinical spectrum of CVID includes not only recurrent infections but also non-infectious manifestations such as pathological lymphoproliferation (lymphadenopathy, splenomegaly), hepatomegaly, interstitial lung disease, inflammatory bowel disease-like syndromes, and autoimmune cytopenias. These features may precede infectious manifestations, complicating both diagnosis and management [7,8].

Secondary AA amyloidosis may also develop in the setting of both an IEI and secondary immunodeficiencies (e.g., HIV infection). According to Gomzikova et al., secondary amyloidosis of the lungs has been observed in patients with cyclic neutropenia, CVID, and hyper-IgM syndrome [9].

Two authors independently searched PubMed and MEDLINE databases for articles reporting amyloidosis in patients with CVID. The search terms included “common variable immunodeficiency”, “CVID”, “amyloidosis”, and “secondary amyloidosis”. Additional publications were identified by manually screening the reference lists of relevant articles. Studies were included if they met the following criteria: written in English, a definite diagnosis of CVID, and clear description of amyloidosis in a CVID patient. Articles containing clinical, laboratory, or histopathological data were considered; duplicate records and non-detailed conference abstracts were excluded. Studies lacking sufficient information on disease progression were also excluded. From each eligible report, the authors extracted the first author’s name, year of publication, patient age and sex, clinical presentation, sites of amyloid deposition, diagnostic methods, treatment, and outcome.

A search for “primary immunodeficiency amyloidosis” in English in international databases (PubMed) for the period from 1962 to 2025 yielded 57 publications, whereas “common variable immunodeficiency secondary amyloidosis” produced less than 10 results.

Table 1 shows the clinical examples described in the literature of patients with AA amyloidosis in combination with an IEI.

The aim of this study is to present a rare clinical case of secondary AA amyloidosis in a patient with an IEI.

## 2. Case Presentation

The patient, a 37-year-old male without burdened family history had a history of recurrent infections since childhood. At the age of 13 (2001) and 15 (2003), he developed pneumonia following episodes of hyperthermia. In 2001, he also presented with a felon of a left-hand finger and sustained a left humeral fracture complicated by osteomyelitis and sepsis. Acute respiratory viral infections occurred no more than twice annually. Since 1994 (age 6), he experienced frequent exacerbations of chronic tonsillitis, for which he underwent tonsillectomy in 2007 (age 19).

In 2021 (age 33), he contracted coronavirus disease (COVID-19) complicated by bilateral multisegmental pneumonia of moderate severity. Discharge records reported leukocytosis of 15.8 × 10^9^/L (reference range: 5–9 × 10^9^/L) and thrombocytosis of 550 × 10^9^/L (180–320 × 10^9^/L) (Tables S1–S3). Three months later, he developed community-acquired pneumonia. Since then, he has experienced recurrent episodes occurring 2–3 times per year, predominantly during autumn and winter, characterized by productive cough with mucopurulent sputum, fever up to 38.5 °C, and radiologically confirmed sinusitis, requiring courses of antibacterial therapy (Figure 1).

Complete blood counts (CBC) in July 2022 and August 2023 revealed normal platelet counts; however, in August 2024, thrombocytosis (663 × 10^9^/L) was noted. Leukocytosis was documented in August 2023 (18.2 × 10^9^/L) and August 2024 (13.5 × 10^9^/L) beyond the exacerbation of the disease. To exclude chronic myeloproliferative neoplasms, molecular genetic testing was performed in November 2024, which revealed no mutations in *JAK2* (Janus kinase 2), *CALR* (calreticulin), or *MPL* (W515L/K in the myeloproliferative leukemia virus oncogene).

In November 2024, during hospitalization in the otolaryngological department at the place of residence, an infundibulotomy was performed to ensure conditions for the outflow of the contents of the maxillary sinus.

In December 2024, he presented to a nephrologist with bilateral lower leg and foot edema. Echocardiography showed preserved global left ventricular systolic function, without regional wall motion abnormalities. Cardiac chambers were not dilated. Right ventricular contractility was preserved with no evidence of right heart overload. Echocardiographic signs of early pulmonary hypertension were noted (systolic pulmonary artery pressure 33 mmHg). Left ventricular ejection fraction was 56%. Laboratory tests revealed hypoproteinemia (38 g/L) and proteinuria (10.69 g/day), consistent with nephrotic syndrome. Complement components C3 and C4 were within normal limits, antinuclear antibodies and anti-neutrophil cytoplasmic antibodies were negative, and anti-PLA2R antibodies were absent. Immunoglobulin levels were: IgG 0.1 g/L (7–16 g/L), IgA 0.01 g/L (0.7–4.0 g/L), IgM 0.28 g/L (0.4–2.3 g/L). The patient received pulse therapy with intravenous methylprednisolone 500 mg/day for 3 days, followed by oral prednisone 60 mg/day with tapering to 6.25 mg/day. Given the hypoproteinemia, hypo-IgM-emia, and recurrent infections, he received a total of 3.75 g of intravenous immunoglobulin (IVIG).

In January 2025, kidney and gastric mucosal biopsies were performed to determine the cause of nephrotic syndrome. Histological evaluation confirmed AA amyloidosis involving the kidneys and stomach. Iliac crest trephine biopsy revealed no pathological changes. Immunohistochemical analysis of serum and urine proteins did not demonstrate monoclonal secretion.

Chest computed tomography (CT) in January 2025 demonstrated mediastinal lymphadenopathy (green arrows), multiple broncho- and bronchiectasis, features of bronchiolitis (highlighted in green circles) (Figure 2A), fibrotic changes including fibroatelectasis (middle lobe of the right lung and lingular segments of the left lung) and coarse fibrotic distortion of the pulmonary architecture (white arrows), as well as infiltrative consolidations (green arrows) (Figure 2B,C).

Ultrasound of the lymph nodes revealed cervical (up to 19 mm, right), axillary (up to 16 mm, left), and inguinal (up to 18 mm, left) lymphadenopathy.

Infectious and granulomatous diseases were excluded: tuberculosis (PCR for *Mycobacterium tuberculosis* DNA—negative), sarcoidosis (serum angiotensin-converting enzyme within reference range), aspergillosis (galactomannan antigen in bronchoalveolar lavage—negative), pneumocystis infection (antigens in bronchoalveolar lavage (BAL)—negative), cytomegalovirus infection (PCR for CMV DNA—negative), and Epstein–Barr virus infection (PCR for EBV DNA—negative).

Positron emission tomography–computed tomography (PET-CT) was conducted in January 2025 (Figure 3).

Empiric therapy with moxifloxacin was initiated, based on chest CT findings and clinical evidence of sinusitis. A follow-up CT scan after 7 days demonstrated positive dynamics, with regression of peribronchial infiltrates in the lower lobes of both lungs. However, due to persistently elevated CRP levels and BAL culture results showing growth of *Haemophilus influenzae* sensitive to ceftriaxone, moxifloxacin was discontinued and therapy with ceftriaxone 2 g daily was initiated. In addition, antiviral therapy with riamilovir was administered, resulting in normalization of body temperature, reduction in cough, and cessation of sputum production. In January 2025, serum immunoglobulin levels were significantly reduced: IgG 0.3 g/L, IgA 0 g/L, IgM 0.40 g/L.

Considering lymphadenopathy, weight loss, amyloidosis, thrombocytosis, and leukocytosis, Castleman disease was suspected. Interleukin-6 (IL-6) was measured at 6.78 pg/mL (reference range: 0–5.90 pg/mL). However, due to a slight increase in lymph nodes, this disease was excluded from the diagnostic search.

Ultrasound of the shoulder soft tissues in February 2025 revealed no additional fluid collections in periarticular tissues or bursae. Mildly decreased echogenicity of the tendon along the anterior joint surface was noted.

Fibrocolonoscopy in February 2025 revealed findings consistent with chronic terminal ileitis. Small bowel biopsy demonstrated chronic inflammation with marked lymphoid hyperplasia. Based on PET-CT results, there was no convincing evidence of chronic osteomyelitis as a cause of AA amyloidosis. Given the presence of bronchiectasis in a young patient, recurrent infections since childhood, and lymphoid hyperplasia of the intestinal wall, an IEI was considered the most likely cause of renal AA amyloidosis.

In February 2025, the patient again developed productive cough with purulent sputum, fever up to 37.9 °C, leukocytosis (28 × 10^9^/L), and elevated CRP (48.15 mg/L). He was rehospitalized for infectious exacerbation and received adjustment of antibacterial therapy.

In March 2025, he was admitted with right-sided community-acquired pneumonia caused by Streptococcus pneumoniae, complicated by bilateral pneumothorax. Treatment included cefotaxime, levofloxacin, fluconazole, and albumin infusions.

Whole-exome sequencing (WES) was recommended. DNA was extracted from peripheral blood using the MGIEasy Universal DNA Library Prep Set (MGI Tech, Shenzhen, China). Sample preparation included ultrasonic fragmentation of genomic DNA (Covaris S220, Woburn, MA, USA) and exonic enrichment with Agilent SureSelect All Exon v8 probes (Agilent Technologies, Santa Clara, CA, USA), according to the laboratory protocol [13]. Sequencing was performed on the G-400 platform (MGI Tech) using paired-end reads. Data processing of fastq files was performed using an automated Python3 pipeline, including quality assessment (FastQC v0.12.1), read trimming (BBDuk v38.96), alignment to the GRCh38/hg38 human reference genome (Bwa MEM2 v2.2.1), SAM-to-BAM conversion and sorting (Samtools v1.9), duplicate marking (Picard v2.22.4), variant calling and normalization (BCFtools v1.9, DeepVariant v1.5.0, vt normalize v0.5772), as well as variant annotation (InterVar v2.2.2) and coverage metrics calculation (Picard v2.22.4) [14]. Variant classification followed ACMG guidelines [15].

Variant filtering was performed using a minor allele frequency (MAF) threshold of <0.01 in gnomAD v4.1.0 (combined genomes and exomes dataset), with a minimum read depth of ≥10× and standard variant-level quality metrics. All candidate variants were manually inspected using the Integrative Genomics Viewer (IGV). In addition to targeted analysis, exome-wide screening was performed for variants annotated as “Pathogenic” or “Likely pathogenic” according to InterVar_conclusion. Sequencing quality metrics demonstrated high coverage, with a median depth of 121×, mean depth of 131.5×, and 97.94%, 97.16%, and 95.92% of target regions covered at ≥10×, ≥20×, and ≥30×, respectively.

A curated panel of 1143 genes associated with primary immunodeficiency, autoinflammatory diseases, hereditary amyloidosis, abnormal immunoglobulin levels, and related immune dysregulation phenotypes was compiled from Genomics England PanelApp, relevant HPO terms, commercial diagnostic panels (Blueprint Genetics and Invitae), and the published literature. The full gene list is provided in Table S4. For the custom gene panel, the mean coverage depth was 130.8×, with 97.21% of target bases covered at ≥30×, ensuring robust variant detection.

Genetic testing (19 March 2025) revealed a previously undescribed likely pathogenic heterozygous variant in the *ODAD2* gene. The variant is located in exon 16 (chr10:27935137G>A) and results in a premature stop codon at position 790 (p.Arg790Ter, NM_018076.5), a known disease-causing mechanism. While biallelic *ODAD2* mutations are associated with primary ciliary dyskinesia (PCD) type 23 (OMIM: 615451), this variant was found in a heterozygous state. This finding is considered incidental and clinically irrelevant to the patient’s phenotype of amyloidosis and CVID. No pathogenic, likely pathogenic, or variants of uncertain significance were identified in genes associated with autoinflammatory diseases, hereditary amyloidosis, IEIs (e.g., agammaglobulinemia), or renal disease.

The patient was admitted to the Department of Immunology, NRC Institute of Immunology, FMBA of Russia in April 2025.

On clinical and immunological examination, persistent leukocytosis (up to 28 × 10^9^/L), thrombocytosis, hypochromic anemia of mixed etiology (iron deficiency and chronic inflammation), toxic granulation of neutrophils, elevated ESR, hypoproteinemia (due to both albumin and globulin deficiency), and increased CRP were noted (Tables S1 and S2). Urinalysis revealed marked proteinuria. Chest CT demonstrated bilateral multisegmental bronchopneumonia, bronchiolitis, mediastinal lymphadenopathy, and bronchiectasis.

Immunological assessment showed panhypogammaglobulinemia with normal total B-lymphocyte counts. Subpopulation analysis revealed a shift toward CD8^+^ lymphocytes, inversion of the CD4^+^/CD8^+^ ratio to 0.47, and a marked reduction in switched memory B cells. Laboratory results are presented in Tables S1–S3 and S5.

Sputum culture revealed growth of *Candida albicans* (10^3^ CFU/mL) and *Pluralibacter gergoviae* (10^3^ CFU/mL, ESBL-producing strain, *Enterobacter gergoviae*).

Differential diagnosis considered an IEI versus secondary immunodeficiency associated with amyloidosis and protein loss. However, given the clinical history (recurrent childhood infections, including osteomyelitis complicated by sepsis; persistent hypoproteinemia since 2021 predating nephrotic syndrome; recurrent sinusitis, bronchitis, multiple pneumonias), radiological findings (bronchiectasis), and immunological abnormalities (severe hypogammaglobulinemia, low switched B-cell counts), a diagnosis of IEI: CVID was established. Additional diagnoses included chronic pansinusitis, chronic otitis media, right-sided sialadenitis, and terminal ileitis.

During the initial hospitalization, differential diagnostics were performed between an IEI and secondary hypogammaglobulinemia due to renal pathology. The patient had complicated infectious history—recurrent pneumonia from childhood, sepsis, hematogenous osteomyelitis, purulent sinusitis, recurrent bronchitis, and bronchiectasis (long before the renal disease). There was also information about low total protein levels (according to medical records, also long before the development of renal amyloidosis), but he was not examined by an immunologist that time. Immunological examination revealed decreased immunoglobulins and very low memory-switched B-lymphocytes (which is not typical for secondary hypogammaglobulinemia).

Taking into account the above, there was more evidence for an IEI with the development of amyloidosis due to uncontrolled infections, rather than SAD. Molecular genetic testing (whole-exome sequencing) did not reveal any pathogenic variants. Whole-genome sequencing is in progress. We did not conduct a post-vaccination response study on him, since the combination of symptoms was sufficient to make a diagnosis. At the time of hospitalization, the patient was in a state of acute infection requiring the initiation of replacement therapy, which did not allow us to leave him without the introduction of immunoglobulin and observe the post-vaccination response after 4–6 weeks.

Lifelong replacement therapy with human normal immunoglobulin at 0.4 g/kg/month (35 g) was recommended. Due to renal disease with protein loss and insufficient IgG trough levels during IVIG therapy, subcutaneous immunoglobulin (SCIG) replacement was preferred.

Considering persistent bronchiectasis results of sputum culture examination, prolonged antibacterial therapy with levofloxacin and fluconazole was administered.

At present, the patient reports satisfactory well-being, without exacerbations of chronic infectious diseases or new acute infections against the background of PCIG for 5 months, the level of inflammatory markers is within the limits of the reference values. The patient is going to return to undergo the examination in May 2026 during which it is planned to measure the serum AA.

## 3. Discussion

In the present case, the initial diagnosis was AA amyloidosis, confirmed by duodenal and renal biopsies. Investigation of the underlying cause of AA amyloidosis revealed CVID, evidenced by laboratory findings, including hypogammaglobulinemia and reduced numbers of class-switched memory B cells (CD19^+^CD27^+^IgD^−^).

The clinical manifestation of CVID occurred between two distinct disease peaks: at the age of 13, the patient developed osteomyelitis complicated by sepsis, an atypical initial presentation of CVID. However, the further course of infectious complications—pneumonia, recurrent sinusitis, as well as a decrease in total protein levels detected at an early stage—should have been the criterion for inclusion in the spectrum of differential diagnosis of IEIs. Delplanque et al. described a similar case of a 24-year-old female with multiple infectious diseases (recurrent influenza, pneumonias, oral herpes, vaginal abscess, meningitis, tubo-ovarian abscess) prior to CVID diagnosis. The patient presented with dyspeptic symptoms, and duodenal and renal biopsies revealed amyloid deposition [16]. These findings underscore the importance of considering an IEI in patients with recurrent infections to prevent severe complications.

In our patient, amyloidosis led to nephrotic syndrome, characterized by massive proteinuria, hypoalbuminemia, and edema. AA amyloidosis often leads to nephrotic syndrome with massive proteinuria and hypoalbuminemia [17]. Secondary immunodeficiency can also develop in nephrotic syndrome due to urinary loss of gamma globulin fractions. In this case, however, laboratory testing confirmed an IEI rather than secondary immunodeficiency, although protein loss contributed to further impairment of humoral immunity.

In the context of AA amyloidosis, patient prognosis progressively worsens, as achieving target pretreatment IgG concentrations is challenging in the presence of continuous protein loss. SCIG is preferable over IVIG, as it reduces renal burden while maintaining a more stable IgG concentration throughout the month.

Lopes et al. reported a case of a 47-year-old female with CVID who developed nephrotic syndrome and acute kidney injury in the setting of recurrent systemic infections caused by *Campylobacter jejuni*. CT imaging demonstrated nephromegaly, and renal biopsy revealed amyloid deposition. Immunofluorescence was negative for IgM, IgG, IgA, C1q, C3c, and kappa/lambda light chains. This case highlights amyloid nephropathy as a cause of acute kidney injury [11]. Our patient also developed amyloid nephropathy, but without progression to severe renal complications.

According to the ESID registry, up to 94% of patients have some kind of lung changes, while the frequency of bronchiectasis, according to data from different centers, varies widely from 0 to 66% [17]. According to Delplanque et al., bronchiectasis should be considered a “red flag” for clinicians, as it represents a source of chronic inflammation that may ultimately contribute to secondary amyloidosis [16].

Rameev et al. (2021) presented data from 110 patients with biopsy-proven AA amyloidosis, of whom 12 (8.96%) had chronic infections [18].

In the differential diagnosis of our case, Castleman disease was considered. This non-clonal lymphoproliferative disorder may present as either unicentric or multicentric lymphadenopathy. Hyperproduction of IL-6 plays a central role in lymphadenopathy and is also associated with pulmonary hypertension typical of Castleman disease. Several clinical variants have been described: the hyaline-vascular type (typically affecting mediastinal lymph nodes), plasma cell type (associated with abdominal lymphadenopathy), multicentric type (with hepatosplenomegaly, pancytopenia, hemolytic anemia, hypoalbuminemia, hypergammaglobulinemia), and HIV-associated multicentric type [19]. In our patient, Castleman disease was excluded due to only mildly elevated IL-6 levels, hypogammaglobulinemia, and absence of pancytopenia. However, biopsy with immunohistochemical analysis of affected lymph nodes would be required for definitive exclusion.

Beyond congenital immunodeficiencies, other causes of recurrent pulmonary infections with early development of bronchiectasis and amyloidosis include cystic fibrosis and PCD. Molecular genetic testing in our patient revealed a likely pathogenic variant in the *ODAD2* gene, which in a homozygous or compound heterozygous state can cause PCD type 23. However, since the variant was identified in the heterozygous state, it represents an incidental finding unrelated to the clinical presentation.

## 4. Conclusions

IEIs, particularly CVID, remain underrecognized in adults presenting with AA amyloidosis and nephrotic syndrome. This case highlights the importance of considering immunological evaluation in patients with recurrent sinopulmonary infections, bronchiectasis, or unexplained chronic inflammation. In the setting of protein-losing nephropathy, maintenance of adequate IgG levels may be challenging, and SCIG may represent a practical alternative to IVIG. Early recognition and initiation of immunoglobulin replacement therapy may help reduce infectious complications and limit further organ damage.

## Figures and Tables

**Figure 1:**
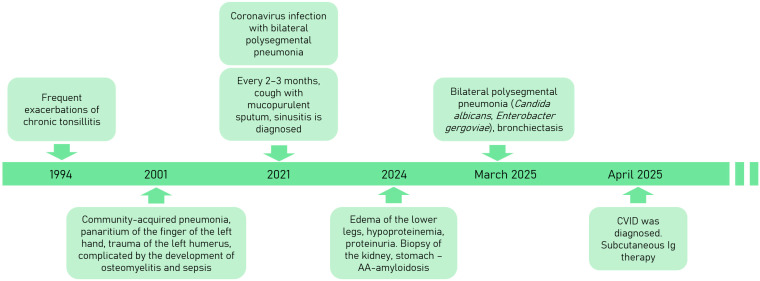
Patient’s timeline.

**Figure 2:**
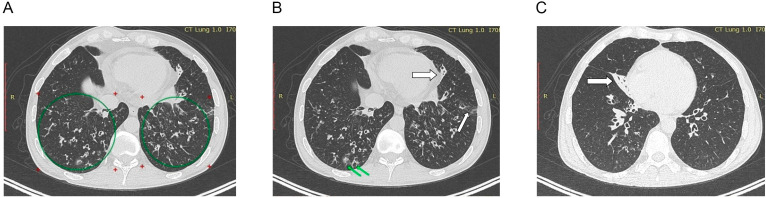
Chest computed tomography scan, January 2025. (**A**) Lung damage in the form of bronchiectasis, bronchiolitis. (**B**,**C**) Lung fibrosis—white arrows, infiltrative consolidation—green arrows.

**Figure 3:**
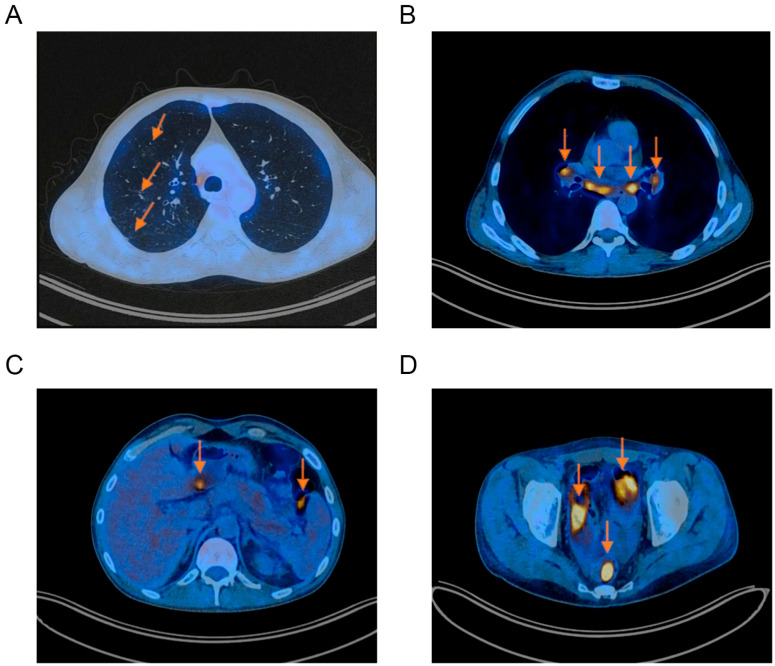
Combined positron emission tomography–computed tomography images. (**A**) Areas of compaction of the lung tissue of the right lung with low radiotracer metabolism, changes of an inflammatory/post-inflammatory nature are more likely. (**B**) Enlarged intrathoracic veins with increased metabolism of radiotracer. (**C**) Site of radiotracer hyperfixation in the walls of the gastric outlet, areas of infiltration of adipose tissue, and a small amount of fluid in the abdominal cavity. (**D**) Sites of radiotracer hyperfixation in the walls of the sigmoid colon and rectosigmoid junction, ascites. Arrows denote the pathological lesion.

**Table 1:** Comparative characteristics of clinical cases of patients with an inborn error of immunity.

	Arslan S. et al. (2015) [10]	Lopes J. et al. (2022) [11]	Kadiroğlu et al. (2012) [12]
Patient’s gender	Male	Female	Female
Age	24 y.o.	47 y.o.	24 y.o.
Nature of the infectious disease	Chronic respiratory infections, bronchiectasis	Recurrent bacteremia caused by microorganisms of the genus *Campylobacter*	Frequent pneumonia
Amyloidosis-affected organs	Lungs (+ pulmonary hypertension)	Kidneys (nephrotic syndrome; molecularly confirmed AA)	Kidneys (nephrotic syndrome)
Diagnostic method	Examination + lung biopsy and CT	Kidney biopsy + serum amyloid A	Kidney biopsy
Amyloid type	Secondary (presumably AA)	AA	AA
Treatment/outcome	Death from septic shock	Hemodialysis, success after Ig correction	Angiotensin-converting enzyme inhibitors, statins, continuation of intravenous immunoglobulin

## Data Availability

Corresponding author upon request.

## Supplementary Materials

The following supporting information can be downloaded at: https://www.mdpi.com/article/10.3390/jcm15114030/s1, Table S1: Laboratory parameters of complete blood count. Table S2: Laboratory parameters of biochemical analysis. Table S3: Immunophenotyping of B lymphocytes. Table S4: Composition of the custom immune gene panel. Table S5: Urinalysis.
