# Supplementary material for: Prolonged Infections and Inflammatory Diseases in Common Variable Immune Deficiency as a Cause of AA Amyloidosis
Source: J Clin Med. 2026 May 22;15(11):4030. doi: 10.3390/jcm15114030 (PMC13257565; doi:10.3390/jcm15114030)
Supplement: Supplementary file 1 [file jcm-15-04030-s001.zip › jcm-4267832-supplementary.pdf]

Supplementary Material

Supplementary Table S1. Laboratory parameters of complete blood count.

**Parameter, units**	**2021 (post-COVID-19 infection)**	**July 2022**	**August 2024**	**November 2024**	**February 2025**	**On admission**	**At discharge**	**Reference range**
Hemoglobin, g/L						109	115	130–160
Erythrocytes, $\times 10^9$/L						4.4	4.44	4–5
Platelets, $\times 10^9$/L	550	344		883	563	728	715	180–320
Leukocytes, $\times 10^9$/L	15.8	18.2	13.5	13.3	36.1	28.1	14.1	4–9
Neutrophils, %<br>Neutrophils, $\times 10^9$/L						70.3<br>19.7	48<br>6.8	42–72<br>2–5.5
Lymphocytes, %<br>Lymphocytes, $\times 10^9$/L						23.4<br>6.58	46.1<br>6.55	19–37<br>1.2–3
Erythrocyte sedimentation rate (ESR), mm/h						85	99	2–15

Supplementary Table S2. Laboratory parameters of biochemical analysis.

**Parameter, units**	**2021 (post-COVID-19 infection)**	**November 2024**	**December 2024**	**February 2025**	**On admission to immunology ward**	**At discharge from immunology ward**	**Reference range**
CRP, mg/L				12.4	44.2	0.7	0–5
Total protein, g/L	52	44.3	38	38.2	35	34	66–83
Albumin, g/L			23		18	18	35–52
Creatinine, µmol/L					105	115	74–110
eGFR CKD-EPI, mL/min/1.73 m^2					78	45	88–128

Immunoglobulin G, g/L					0.87	2.68	9–18
Immunoglobulin A, g/L					0.01		1–3.5
Immunoglobulin M, g/L					0.25		9–18

Note: CRP – C-reactive protein; eGFR – estimated glomerular filtration rate; CKD-EPI – Chronic Kidney Disease Epidemiology Collaboration equation.

Supplementary Table S3. Immunophenotyping of B lymphocytes.

**Parameter**	**Result**	**Reference range**
Lymphocytes (per µL of blood)	6575	1200–3000
$CD3^{+}$ T cells (% of lymphocytes)	67.5	55–80
$CD3^{+}$ T cells (per µL)	4438	800–2200
$CD3^{+}CD4^{+}$ T helper cells (% of lymphocytes)	21.1	31–49
$CD3^{+}CD4^{+}$ T helper cells (per µL)	1387	600–1600
$CD3^{+}CD8^{+}$ cytotoxic T cells (% of lymphocytes)	44.7	12–30
$CD3^{+}CD8^{+}$ cytotoxic T cells (per µL)	2939	190–650
$CD3^{+}CD4^{+}/CD3^{+}CD8^{+}$ ratio	0.47	1.5–3.0
$CD3^{+}CD4^{+}CD8^{+}$ (% of lymphocytes)	0.4	<2
$CD3^{+}CD4^{-}CD8^{-}$ (% of lymphocytes)	2.1	–
$CD3^{-}CD16,56^{+}$ NK cells (% of lymphocytes)	22.5	6–20
$CD3^{-}$CD16,56+ NK cells (per µL)	1479	150–600
$CD3^{+}CD16,56^{+}$ cells (% of lymphocytes)	7.8	<10
$CD3^{+}CD16,56^{+}$ cells (per µL)	513	0–300
$CD19^{+}$ B cells (% of lymphocytes)	8.2	5–19
$CD19^{+}$ B cells (per µL)	539	100–500

IgD^{+}CD27$^{–}$ naive B cells (% of B cells)	85.1	43–82*
IgM^{+}CD27$^{–}$ naive B cells (% of B cells)	90.5	43–82*
IgD^{+}CD27+ marginal zone B cells (% of B cells)	7.4	7.5–32.5*
IgM^{+}CD27^{+} marginal zone B cells (% of B cells)	6.4	7.5–32.5*
IgD$^{–}$CD27^{+} switched memory B cells (% of B cells)	3.0	6.5–29*
IgM$^{–}$CD27^{+} switched memory B cells (% of B cells)	1.3	6.5–29*
IgD^{+}CD38^{++} transitional B cells (% of B cells)	3.7	0.6–3.4*
IgM^{+}CD38^{++} transitional B cells (% of B cells)	3.3	0.6–3.4*
IgD$^{–}$CD38^{+++} plasmablasts (% of B cells)	0.3	0.4–3.6*
IgM$^{–}$CD38^{+++} plasmablasts (% of B cells)	0.1	0.4–3.6*
CD21^{low}CD38$^{–}$ activated B cells (% of B cells)	20.1	0.9–7.6*
BAFF-R^{+} cells (% of B cells)	99.8	>95%

Supplementary Table S4. Composition of the custom immune gene panel.

**Gene**	**Name**	**HGNC ID**	**Location**
A2ML1	alpha-2-macroglobulin like 1	HGNC:23336	12p13.31
AARS1	alanyl-tRNA synthetase 1	HGNC:20	16q22.1
ABCB11	ATP binding cassette subfamily B member 11	HGNC:42	2q31.1
ABCB4	ATP binding cassette subfamily B member 4	HGNC:45	7q21.12
ABCB7	ATP binding cassette subfamily B member 7	HGNC:48	Xq13.3
ABCC8	ATP binding cassette subfamily C member 8	HGNC:59	11p15.1
ABCD4	ATP binding cassette subfamily D member 4	HGNC:68	14q24.3
ABCG5	ATP binding cassette subfamily G member 5	HGNC:13886	2p21
ABCG8	ATP binding cassette subfamily G member 8	HGNC:13887	2p21
ABI3	ABI family member 3	HGNC:29859	17q21.32
ACAN	aggrecan	HGNC:319	15q26.1
ACD	ACD shelterin complex subunit and telomerase recruitment factor	HGNC:25070	16q22.1
ACP5	acid phosphatase 5, tartrate resistant	HGNC:124	19p13.2
ACTB	actin beta	HGNC:132	7p22.1
ACTG1	actin gamma 1	HGNC:144	17q25.3
ACTN1	actinin alpha 1	HGNC:163	14q24.1
ADA	adenosine deaminase	HGNC:186	20q13.12
ADA2	adenosine deaminase 2	HGNC:1839	22q11.1
ADAM17	ADAM metallopeptidase domain 17	HGNC:195	2p25.1

ADAMTS13	ADAM metallopeptidase with thrombospondin type 1 motif 13	HGNC:1366	9q34.2
ADAMTS3	ADAM metallopeptidase with thrombospondin type 1 motif 3	HGNC:219	4q13.3
ADAR	adenosine deaminase RNA specific	HGNC:225	1q21.3
ADGRE2	adhesion G protein-coupled receptor E2	HGNC:3337	19p13.12
ADIPOQ	adiponectin, C1Q and collagen domain containing	HGNC:13633	3q27.3
ADIPOR1	adiponectin receptor 1	HGNC:24040	1q32.1
ADIPOR2	adiponectin receptor 2	HGNC:24041	12p13.33
AGA	aspartylglucosaminidase	HGNC:318	4q34.3
AGL	amylo-alpha-1,6-glucosidase and 4-alpha-glucanotransferase	HGNC:321	1p21.2
AGR2	anterior gradient 2, protein disulphide isomerase family member	HGNC:328	7p21.1
AGRN	agrin	HGNC:329	1p36.33
AICDA	activation induced cytidine deaminase	HGNC:13203	12p13.31
AIRE	autoimmune regulator	HGNC:360	21q22.3
AK2	adenylate kinase 2	HGNC:362	1p35.1
AK7	adenylate kinase 7	HGNC:20091	14q32.2
AKT1	AKT serine/threonine kinase 1	HGNC:391	14q32.33
AKT2	AKT serine/threonine kinase 2	HGNC:392	19q13.2
ALAS2	5'-aminolevulinate synthase 2	HGNC:397	Xp11.21
ALB	albumin	HGNC:399	4q13.3
ALG1	ALG1 chitobiosyldiphosphodolichol beta-mannosyltransferase	HGNC:18294	16p13.3
ALG12	ALG12 alpha-1,6-mannosyltransferase	HGNC:19358	22q13.33
ALG14	ALG14 UDP-N-acetylglucosaminyltransferase subunit	HGNC:28287	1p21.3
ALG6	ALG6 alpha-1,3-glucosyltransferase	HGNC:23157	1p31.3
ALPI	alkaline phosphatase, intestinal	HGNC:437	2q37.1
ALPK1	alpha kinase 1	HGNC:20917	4q25
AMFR	autocrine motility factor receptor	HGNC:463	16q13
AMN	amnion associated transmembrane protein	HGNC:14604	14q32.32
ANGPT1	angiopoietin 1	HGNC:484	8q23.1
ANKRD11	ankyrin repeat domain 11	HGNC:21316	16q24.3
ANKRD26	ankyrin repeat domain 26	HGNC:29186	10p12.1
ANKRD55	ankyrin repeat domain 55	HGNC:25681	5q11.2
ANKZF1	ankyrin repeat and zinc finger peptidyl tRNA hydrolase 1	HGNC:25527	2q35
ANO6	anoctamin 6	HGNC:25240	12q12
ANTXR2	ANTXR cell adhesion molecule 2	HGNC:21732	4q21.21
AP1S3	adaptor related protein complex 1 subunit sigma 3	HGNC:18971	2q36.1
AP3B1	adaptor related protein complex 3 subunit beta 1	HGNC:566	5q14.1
AP3D1	adaptor related protein complex 3 subunit delta 1	HGNC:568	19p13.3
APOA1	apolipoprotein A1	HGNC:600	11q23.3
APOA2	apolipoprotein A2	HGNC:601	1q23.3
APOC2	apolipoprotein C2	HGNC:609	19q13.32
APOC3	apolipoprotein C3	HGNC:610	11q23.3
APOL1	apolipoprotein L1	HGNC:618	22q12.3
APP	amyloid beta precursor protein	HGNC:620	21q21.3
APPL1	adaptor protein, phosphotyrosine interacting with PH domain and leucine zipper 1	HGNC:24035	3p14.3
ARHGAP42	Rho GTPase activating protein 42	HGNC:26545	11q22.1
ARHGEF1	Rho guanine nucleotide exchange factor 1	HGNC:681	19q13.2
ARPC1B	actin related protein 2/3 complex subunit 1B	HGNC:704	7q22.1
ARPC5	actin related protein 2/3 complex subunit 5	HGNC:708	1q25.3

ARVCF	ARVCF delta catenin family member	HGNC:728	22q11.21
ASAH1	N-acylsphingosine amidohydrolase 1	HGNC:735	8p22
ASXL1	ASXL transcriptional regulator 1	HGNC:18318	20q11.21
ATAD3A	ATPase family AAA domain containing 3A	HGNC:25567	1p36.33
ATG4A	autophagy related 4A cysteine peptidase	HGNC:16489	Xq22.3
ATM	ATM serine/threonine kinase	HGNC:795	11q22.3
ATP6AP1	ATPase H+ transporting accessory protein 1	HGNC:868	Xq28
ATP6AP2	ATPase H+ transporting accessory protein 2	HGNC:18305	Xp11.4
ATP8B1	ATPase phospholipid transporting 8B1	HGNC:3706	18q21.31
ATR	ATR checkpoint kinase	HGNC:882	3q23
ATRX	ATRX chromatin remodeler	HGNC:886	Xq21.1
AURKB	aurora kinase B	HGNC:11390	17p13.1
B2M	beta-2-microglobulin	HGNC:914	15q21.1
BACH2	BACH transcriptional regulator 2	HGNC:14078	6q15
BANK1	B cell scaffold protein with ankyrin repeats 1	HGNC:18233	4q24
BCL10	BCL10 immune signaling adaptor	HGNC:989	1p22.3
BCL11B	BCL11 transcription factor B	HGNC:13222	14q32.2
BCO1	beta-carotene oxygenase 1	HGNC:13815	16q23.2
BCR	BCR activator of RhoGEF and GTPase	HGNC:1014	22q11.23
BLK	BLK proto-oncogene, Src family tyrosine kinase	HGNC:1057	8p23.1
BLM	BLM RecQ like helicase	HGNC:1058	15q26.1
BLNK	B cell linker	HGNC:14211	10q24.1
BLOC1S3	biogenesis of lysosomal organelles complex 1 subunit 3	HGNC:20914	19q13.32
BLOC1S6	biogenesis of lysosomal organelles complex 1 subunit 6	HGNC:8549	15q21.1
BRAF	B-Raf proto-oncogene, serine/threonine kinase	HGNC:1097	7q34
BRCA1	BRCA1 DNA repair associated	HGNC:1100	17q21.31
BRCA2	BRCA2 DNA repair associated	HGNC:1101	13q13.1
BRIP1	BRCA1 interacting DNA helicase 1	HGNC:20473	17q23.2
BTK	Bruton tyrosine kinase	HGNC:1133	Xq22.1
BUB1B	BUB1 mitotic checkpoint serine/threonine kinase B	HGNC:1149	15q15.1
C1GALT1C1	C1GALT1 specific chaperone 1	HGNC:24338	Xq24
C1QA	complement C1q A chain	HGNC:1241	1p36.12
C1QB	complement C1q B chain	HGNC:1242	1p36.12
C1QBP	complement C1q binding protein	HGNC:1243	17p13.2
C1QC	complement C1q C chain	HGNC:1245	1p36.12
C1R	complement C1r	HGNC:1246	12p13.31
C1S	complement C1s	HGNC:1247	12p13.31
C2	complement C2	HGNC:1248	6p21.33
C2orf69	chromosome 2 open reading frame 69	HGNC:26799	2q33.1
C3	complement C3	HGNC:1318	19p13.3
C3AR1	complement C3a receptor 1	HGNC:1319	12p13.31
C4A	complement C4A (Chido/Rodgers blood group)	HGNC:1323	6p21.33
C4B	complement C4B (Chido/Rodgers blood group)	HGNC:1324	6p21.33
C4BPA	complement component 4 binding protein alpha	HGNC:1325	1q32.2
C4BPB	complement component 4 binding protein beta	HGNC:1328	1q32.1
C5	complement C5	HGNC:1331	9q33.2
C5AR1	complement C5a receptor 1	HGNC:1338	19q13.32
C5AR2	complement C5a receptor 2	HGNC:4527	19q13.32

C6	complement C6	HGNC:1339	5p13.1
C7	complement C7	HGNC:1346	5p13.1
C8A	complement C8 alpha chain	HGNC:1352	1p32.2
C8B	complement C8 beta chain	HGNC:1353	1p32.2
C8G	complement C8 gamma chain	HGNC:1354	9q34.3
C9	complement C9	HGNC:1358	5p13.1
CA2	carbonic anhydrase 2	HGNC:1373	8q21.2
CACNA1C	calcium voltage-gated channel subunit alpha1 C	HGNC:1390	12p13.33
CALR	calreticulin	HGNC:1455	19p13.13
CARD10	caspase recruitment domain family member 10	HGNC:16422	22q13.1
CARD11	caspase recruitment domain family member 11	HGNC:16393	7p22.2
CARD14	caspase recruitment domain family member 14	HGNC:16446	17q25.3
CARD8	caspase recruitment domain family member 8	HGNC:17057	19q13.33
CARD9	caspase recruitment domain family member 9	HGNC:16391	9q34.3
CARMIL2	capping protein regulator and myosin 1 linker 2	HGNC:27089	16q22.1
CARS1	cysteinyl-tRNA synthetase 1	HGNC:1493	11p15.4
CASP10	caspase 10	HGNC:1500	2q33.1
CASP8	caspase 8	HGNC:1509	2q33.1
CAV1	caveolin 1	HGNC:1527	7q31.2
CAVIN1	caveolae associated protein 1	HGNC:9688	17q21.2
CBL	Cbl proto-oncogene	HGNC:1541	11q23.3
CBLB	Cbl proto-oncogene B	HGNC:1542	3q13.11
CBS	cystathionine beta-synthase	HGNC:1550	21q22.3
CCBE1	collagen and calcium binding EGF domains 1	HGNC:29426	18q21.32
CCDC103	dynein axonemal assembly factor 19	HGNC:32700	17q21.31
CCDC39	coiled-coil domain 39 molecular ruler complex subunit	HGNC:25244	3q26.33
CCDC40	coiled-coil domain 40 molecular ruler complex subunit	HGNC:26090	17q25.3
CCDC47	coiled-coil domain containing 47	HGNC:24856	17q23.3
CCDC65	dynein regulatory complex subunit 2	HGNC:29937	12q13.12
CCN2	cellular communication network factor 2	HGNC:2500	6q23.2
CCN6	cellular communication network factor 6	HGNC:12771	6q21
CCND1	cyclin D1	HGNC:1582	11q13.3
CCNK	cyclin K	HGNC:1596	14q32.2
CCNO	cyclin O	HGNC:18576	5q11.2
CCR6	C-C motif chemokine receptor 6	HGNC:1607	6q27
CD19	CD19 molecule	HGNC:1633	16p11.2
CD244	CD244 molecule	HGNC:18171	1q23.3
CD247	CD247 molecule	HGNC:1677	1q24.2
CD27	CD27 molecule	HGNC:11922	12p13.31
CD274	CD274 molecule	HGNC:17635	9p24.1
CD28	CD28 molecule	HGNC:1653	2q33.2
CD3D	CD3 delta subunit of T-cell receptor complex	HGNC:1673	11q23.3
CD3E	CD3 epsilon subunit of T-cell receptor complex	HGNC:1674	11q23.3
CD3G	CD3 gamma subunit of T-cell receptor complex	HGNC:1675	11q23.3
CD4	CD4 molecule	HGNC:1678	12p13.31
CD40	CD40 molecule	HGNC:11919	20q13.12
CD40LG	CD40 ligand	HGNC:11935	Xq26.3
CD46	CD46 molecule	HGNC:6953	1q32.2

CD48	CD48 molecule	HGNC:1683	1q23.3
CD55	CD55 molecule (Cromer blood group)	HGNC:2665	1q32.2
CD59	CD59 molecule (CD59 blood group)	HGNC:1689	11p13
CD70	CD70 molecule	HGNC:11937	19p13.3
CD79A	CD79a molecule	HGNC:1698	19q13.2
CD79B	CD79b molecule	HGNC:1699	17q23.3
CD80	CD80 molecule	HGNC:1700	3q13.33
CD81	CD81 molecule	HGNC:1701	11p15.5
CD8A	CD8 subunit alpha	HGNC:1706	2p11.2
CD93	CD93 molecule	HGNC:15855	20p11.21
CDAN1	codanin 1	HGNC:1713	15q15.2
CDC42	cell division cycle 42	HGNC:1736	1p36.12
CDCA7	cell division cycle associated 7	HGNC:14628	2q31.1
CDH23	cadherin related 23	HGNC:13733	10q22.1
CDK9	cyclin dependent kinase 9	HGNC:1780	9q34.11
CDKN1B	cyclin dependent kinase inhibitor 1B	HGNC:1785	12p13.1
CDKN2A	cyclin dependent kinase inhibitor 2A	HGNC:1787	9p21.3
CDSN	corneodesmosin	HGNC:1802	6p21.33
CEBPA	CCAAT enhancer binding protein alpha	HGNC:1833	19q13.11
CEBPE	CCAAT enhancer binding protein epsilon	HGNC:1836	14q11.2
CEL	carboxyl ester lipase	HGNC:1848	9q34.13
CENPF	centromere protein F	HGNC:1857	1q41
CEP164	centrosomal protein 164	HGNC:29182	11q23.3
CFAP298	cilia and flagella associated protein 298	HGNC:1301	21q22.11
CFB	complement factor B	HGNC:1037	6p21.33
CFD	complement factor D	HGNC:2771	19p13.3
CFH	complement factor H	HGNC:4883	1q31.3
CFHR1	complement factor H related 1	HGNC:4888	1q31.3
CFHR2	complement factor H related 2	HGNC:4890	1q31.3
CFHR3	complement factor H related 3	HGNC:16980	1q31.3
CFHR4	complement factor H related 4	HGNC:16979	1q31.3
CFHR5	complement factor H related 5	HGNC:24668	1q31.3
CFI	complement factor I	HGNC:5394	4q25
CFP	complement factor properdin	HGNC:8864	Xp11.23
CFTR	CF transmembrane conductance regulator	HGNC:1884	7q31.2
CHAT	choline O-acetyltransferase	HGNC:1912	10q11.23
CHD1	chromodomain helicase DNA binding protein 1	HGNC:1915	5q15-q21.1
CHD7	chromodomain helicase DNA binding protein 7	HGNC:20626	8q12.2
CHEK2	checkpoint kinase 2	HGNC:16627	22q12.1
CHRNA1	cholinergic receptor nicotinic alpha 1 subunit	HGNC:1955	2q31.1
CHRNB1	cholinergic receptor nicotinic beta 1 subunit	HGNC:1961	17p13.1
CHRND	cholinergic receptor nicotinic delta subunit	HGNC:1965	2q37.1
CHRNE	cholinergic receptor nicotinic epsilon subunit	HGNC:1966	17p13.2
CHUK	component of inhibitor of nuclear factor kappa B kinase complex	HGNC:1974	10q24.31
CIB1	calcium and integrin binding 1	HGNC:16920	15q26.1
CIITA	class II major histocompatibility complex transactivator	HGNC:7067	16p13.13
CISD2	CDGSH iron sulfur domain 2	HGNC:24212	4q24
CLCN7	Cl-/H+ antiporter 7	HGNC:2025	16p13.3

CLCNKB	chloride voltage-gated channel Kb	HGNC:2027	1p36.13
CLEC7A	C-type lectin domain containing 7A	HGNC:14558	12p13.2
CLPB	ClpB family mitochondrial disaggregase	HGNC:30664	11q13.4
CLU	clusterin	HGNC:2095	8p21.1
CNBP	CCHC-type zinc finger nucleic acid binding protein	HGNC:13164	3q21.3
COG6	component of oligomeric golgi complex 6	HGNC:18621	13q14.11
COL13A1	collagen type XIII alpha 1 chain	HGNC:2190	10q22.1
COL1A1	collagen type I alpha 1 chain	HGNC:2197	17q21.33
COL2A1	collagen type II alpha 1 chain	HGNC:2200	12q13.11
COL4A5	collagen type IV alpha 5 chain	HGNC:2207	Xq22.3
COL4A6	collagen type IV alpha 6 chain	HGNC:2208	Xq22.3
COL7A1	collagen type VII alpha 1 chain	HGNC:2214	3p21.31
COLEC11	collectin subfamily member 11	HGNC:17213	2p25.3
COLQ	collagen like tail subunit of asymmetric acetylcholinesterase	HGNC:2226	3p25.1
COMT	catechol-O-methyltransferase	HGNC:2228	22q11.21
COPA	coat protein complex I subunit alpha	HGNC:2230	1q23.2
COPG1	coat protein complex I subunit gamma 1	HGNC:2236	3q21.3
COPZ1	coat protein complex I subunit zeta 1	HGNC:2243	12q13.13
CORIN	corin, serine peptidase	HGNC:19012	4p12
CORO1A	coronin 1A	HGNC:2252	16p11.2
CPLX1	complexin 1	HGNC:2309	4p16.3
CPT2	carnitine palmitoyltransferase 2	HGNC:2330	1p32.3
CR2	complement C3d receptor 2	HGNC:2336	1q32.2
CRACR2A	calcium release activated channel regulator 2A	HGNC:28657	12p13.32
CREBBP	CREB binding lysine acetyltransferase	HGNC:2348	16p13.3
CRKL	CRK like proto-oncogene, adaptor protein	HGNC:2363	22q11.21
CRP	C-reactive protein	HGNC:2367	1q23.2
CRYAB	crystallin alpha B	HGNC:2389	11q23.1
CSF2	colony stimulating factor 2	HGNC:2434	5q31.1
CSF2RA	colony stimulating factor 2 receptor subunit alpha	HGNC:2435	Xp22.32 and Yp11.3
CSF2RB	colony stimulating factor 2 receptor subunit beta	HGNC:2436	22q12.3
CSF3R	colony stimulating factor 3 receptor	HGNC:2439	1p34.3
CSNK2A1	casein kinase 2 alpha 1	HGNC:2457	20p13
CST3	cystatin C	HGNC:2475	20p11.21
CTBP1	C-terminal binding protein 1	HGNC:2494	4p16.3
CTC1	CST telomere replication complex component 1	HGNC:26169	17p13.1
CTLA4	cytotoxic T-lymphocyte associated protein 4	HGNC:2505	2q33.2
CTNNB1	catenin beta 1	HGNC:2514	3p22.1
CTNNBL1	catenin beta like 1	HGNC:15879	20q11.23
CTPS1	CTP synthase 1	HGNC:2519	1p34.2
CTSC	cathepsin C	HGNC:2528	11q14.2
CUBN	cubilin	HGNC:2548	10p13
CUL4B	cullin 4B	HGNC:2555	Xq24
CXCR2	C-X-C motif chemokine receptor 2	HGNC:6027	2q35
CXCR4	C-X-C motif chemokine receptor 4	HGNC:2561	2q22.1
CYBA	cytochrome b-245 alpha chain	HGNC:2577	16q24.2
CYBB	cytochrome b-245 beta chain	HGNC:2578	Xp21.1-p11.4
CYBC1	cytochrome b-245 chaperone 1	HGNC:28672	17q25.3

CYCS	cytochrome c, somatic	HGNC:19986	7p15.3
CYP27A1	cytochrome P450 family 27 subfamily A member 1	HGNC:2605	2q35
DBF4	DBF4-CDC7 kinase regulatory subunit	HGNC:17364	7q21.12
DBR1	debranching RNA lariats 1	HGNC:15594	3q22.3
DCLRE1B	DNA cross-link repair 1B	HGNC:17641	1p13.2
DCLRE1C	DNA cross-link repair 1C	HGNC:17642	10p13
DDX11	DEAD/H-box helicase 11	HGNC:2736	12p11.21
DDX41	DEAD-box helicase 41	HGNC:18674	5q35.3
DDX58	RNA sensor RIG-I	HGNC:19102	9p21.1
DEF6	DEF6 guanine nucleotide exchange factor	HGNC:2760	6p21.31
DGAT1	diacylglycerol O-acyltransferase 1	HGNC:2843	8q24.3
DGKE	diacylglycerol kinase epsilon	HGNC:2852	17q22
DHFR	dihydrofolate reductase	HGNC:2861	5q14.1
DIAPH1	diaphanous related formin 1	HGNC:2876	5q31.3
DIPK2B	divergent protein kinase domain 2B	HGNC:25866	Xp11.3
DKC1	dyskerin pseudouridine synthase 1	HGNC:2890	Xq28
DNAAF1	dynein axonemal assembly factor 1	HGNC:30539	16q24.1
DNAAF2	dynein axonemal assembly factor 2	HGNC:20188	14q21.3
DNAAF3	dynein axonemal assembly factor 3	HGNC:30492	19q13.42
DNAAF4	dynein axonemal assembly factor 4	HGNC:21493	15q21.3
DNAAF5	dynein axonemal assembly factor 5	HGNC:26013	7p22.3
DNAH1	dynein axonemal heavy chain 1	HGNC:2940	3p21.1
DNAH11	dynein axonemal heavy chain 11	HGNC:2942	7p15.3
DNAH5	dynein axonemal heavy chain 5	HGNC:2950	5p15.2
DNAH8	dynein axonemal heavy chain 8	HGNC:2952	6p21.2
DNAH9	dynein axonemal heavy chain 9	HGNC:2953	17p12
DNAI1	dynein axonemal intermediate chain 1	HGNC:2954	9p13.3
DNAI2	dynein axonemal intermediate chain 2	HGNC:18744	17q25.1
DNAJB13	DnaJ heat shock protein family (Hsp40) member B13	HGNC:30718	11q13.4
DNAJC21	DnaJ heat shock protein family (Hsp40) member C21	HGNC:27030	5p13.2
DNAJC3	DnaJ heat shock protein family (Hsp40) member C3	HGNC:9439	13q32.1
DNAL1	dynein axonemal light chain 1	HGNC:23247	14q24.3
DNASE1	deoxyribonuclease 1	HGNC:2956	16p13.3
DNASE1L3	deoxyribonuclease 1L3	HGNC:2959	3p14.3
DNASE2	deoxyribonuclease 2, lysosomal	HGNC:2960	19p13.13
DNMT3B	DNA methyltransferase 3 beta	HGNC:2979	20q11.21
DOCK11	dedicator of cytokinesis 11	HGNC:23483	Xq24
DOCK2	dedicator of cytokinesis 2	HGNC:2988	5q35.1
DOCK8	dedicator of cytokinesis 8	HGNC:19191	9p24.3
DOK7	docking protein 7	HGNC:26594	4p16.3
DPAGT1	dolichyl-phosphate N-acetylglucosaminephosphotransferase 1	HGNC:2995	11q23.3
DPP9	dipeptidyl peptidase 9	HGNC:18648	19p13.3
DRC1	dynein regulatory complex subunit 1	HGNC:24245	2p23.3
DSG1	desmoglein 1	HGNC:3048	18q12.1
DTNBP1	dystrobrevin binding protein 1	HGNC:17328	6p22.3
DUOX2	dual oxidase 2	HGNC:13273	15q21.1
DUT	deoxyuridine triphosphatase	HGNC:3078	15q21.1
EDA	ectodysplasin A	HGNC:3157	Xq13.1

EDAR	ectodysplasin A receptor	HGNC:2895	2q13
EDARADD	EDAR associated via death domain	HGNC:14341	1q42.3-q43
EFL1	elongation factor like GTPase 1	HGNC:25789	15q25.2
EGFR	epidermal growth factor receptor	HGNC:3236	7p11.2
EIF2AK3	eukaryotic translation initiation factor 2 alpha kinase 3	HGNC:3255	2p11.2
EIF2AK4	eukaryotic translation initiation factor 2 alpha kinase 4	HGNC:19687	15q15.1
EIF6	eukaryotic translation initiation factor 6	HGNC:6159	20q11.22
ELANE	elastase, neutrophil expressed	HGNC:3309	19p13.3
ELF4	E74 like ETS transcription factor 4	HGNC:3319	Xq26.1
ELP1	elongator acetyltransferase complex subunit 1	HGNC:5959	9q31.3
EP300	EP300 lysine acetyltransferase	HGNC:3373	22q13.2
EPCAM	epithelial cell adhesion molecule	HGNC:11529	2p21
EPG5	ectopic P-granules 5 autophagy tethering factor	HGNC:29331	18q12.3-q21.1
EPO	erythropoietin	HGNC:3415	7q22.1
ERBIN	erbb2 interacting protein	HGNC:15842	5q12.3
ERCC2	ERCC excision repair 2, TFIIH core complex helicase subunit	HGNC:3434	19q13.32
ERCC3	ERCC excision repair 3, TFIIH core complex helicase subunit	HGNC:3435	2q14.3
ERCC4	ERCC excision repair 4, endonuclease catalytic subunit	HGNC:3436	16p13.12
ERCC6L2	ERCC excision repair 6 like 2	HGNC:26922	9q22.32
ETS1	ETS proto-oncogene 1, transcription factor	HGNC:3488	11q24.3
ETV6	ETS variant transcription factor 6	HGNC:3495	12p13.2
EXTL3	exostosin like glycosyltransferase 3	HGNC:3518	8p21.1
EZR	ezrin	HGNC:12691	6q25.3
F12	coagulation factor XII	HGNC:3530	5q35.3
FAAP100	FA core complex associated protein 100	HGNC:26171	17q25.3
FAAP24	FA core complex associated protein 24	HGNC:28467	19q13.11
FADD	Fas associated via death domain	HGNC:3573	11q13.3
FAM111B	FAM111 trypsin like peptidase B	HGNC:24200	11q12.1
FANCA	FA complementation group A	HGNC:3582	16q24.3
FANCB	FA complementation group B	HGNC:3583	Xp22.2
FANCC	FA complementation group C	HGNC:3584	9q22.32
FANCD2	FA complementation group D2	HGNC:3585	3p25.3
FANCE	FA complementation group E	HGNC:3586	6p21.31
FANCF	FA complementation group F	HGNC:3587	11p14.3
FANCG	FA complementation group G	HGNC:3588	9p13.3
FANCI	FA complementation group I	HGNC:25568	15q26.1
FANCL	FA complementation group L	HGNC:20748	2p16.1
FANCM	FA complementation group M	HGNC:23168	14q21.2
FAS	Fas cell surface death receptor	HGNC:11920	10q23.31
FASLG	Fas ligand	HGNC:11936	1q24.3
FAT4	FAT atypical cadherin 4	HGNC:23109	4q28.1
FBF1	Fas binding factor 1	HGNC:24674	17q25.1
FBN1	fibrillin 1	HGNC:3603	15q21.1
FBRS	fibrosin	HGNC:20442	16p11.2
FCGR1A	Fc gamma receptor Ia	HGNC:3613	1q21.2
FCGR2A	Fc gamma receptor IIa	HGNC:3616	1q23.3
FCGR2B	Fc gamma receptor IIb	HGNC:3618	1q23.3
FCGR2C	Fc gamma receptor IIc (gene/pseudogene)	HGNC:15626	1q23.3

FCGR3A	Fc gamma receptor IIIa	HGNC:3619	1q23.3
FCGR3B	Fc gamma receptor IIIb	HGNC:3620	1q23.3
FCGRT	Fc gamma receptor and transporter	HGNC:3621	19q13.33
FCHO1	FCH and mu domain containing endocytic adaptor 1	HGNC:29002	19p13.11
FCN1	ficolin 1	HGNC:3623	9q34.3
FCN2	ficolin 2	HGNC:3624	9q34.3
FCN3	ficolin 3	HGNC:3625	1p36.11
FECH	ferrochelatase	HGNC:3647	18q21.31
FERMT1	FERM domain containing kindlin 1	HGNC:15889	20p12.3
FERMT3	FERM domain containing kindlin 3	HGNC:23151	11q13.1
FGA	fibrinogen alpha chain	HGNC:3661	4q31.3
FGFRL1	fibroblast growth factor receptor like 1	HGNC:3693	4p16.3
FGL2	fibrinogen like 2	HGNC:3696	7q11.23
FLG	filaggrin	HGNC:3748	1q21.3
FLI1	Fli-1 proto-oncogene, ETS transcription factor	HGNC:3749	11q24.3
FLNA	filamin A	HGNC:3754	Xq28
FLT1	fms related receptor tyrosine kinase 1	HGNC:3763	13q12.3
FLT3LG	fms related receptor tyrosine kinase 3 ligand	HGNC:3766	19q13.33
FMNL2	formin like 2	HGNC:18267	2q23.3
FMR1	fragile X messenger ribonucleoprotein 1	HGNC:3775	Xq27.3
FNIP1	folliculin interacting protein 1	HGNC:29418	5q31.1
FOXD3	forkhead box D3	HGNC:3804	1p31.3
FOXI3	forkhead box I3	HGNC:35123	2p11.2
FOXM1	forkhead box M1	HGNC:3818	12p13.33
FOXN1	forkhead box N1	HGNC:12765	17q11.2
FOXP3	forkhead box P3	HGNC:6106	Xp11.23
FPR1	formyl peptide receptor 1	HGNC:3826	19q13.41
FPR2	formyl peptide receptor 2	HGNC:3827	19q13.41
FPR3	formyl peptide receptor 3	HGNC:3828	19q13.41
FRAS1	Fraser extracellular matrix complex subunit 1	HGNC:19185	4q21.21
G6PC3	glucose-6-phosphatase catalytic subunit 3	HGNC:24861	17q21.31
G6PD	glucose-6-phosphate dehydrogenase	HGNC:4057	Xq28
GAD1	glutamate decarboxylase 1	HGNC:4092	2q31.1
GALC	galactosylceramidase	HGNC:4115	14q31.3
GAS2L2	growth arrest specific 2 like 2	HGNC:24846	17q12
GAS8	dynein regulatory complex subunit 4	HGNC:4166	16q24.3
GATA1	GATA binding protein 1	HGNC:4170	Xp11.23
GATA2	GATA binding protein 2	HGNC:4171	3q21.3
GBA	glucosylceramidase beta 1	HGNC:4177	1q22
GCC2	GRIP and coiled-coil domain containing 2	HGNC:23218	2q12.3
GCK	glucokinase	HGNC:4195	7p13
GFI1	growth factor independent 1 transcriptional repressor	HGNC:4237	1p22.1
GFI1B	growth factor independent 1B transcriptional repressor	HGNC:4238	9q34.13
GIMAP5	GTPase, IMAP family member 5	HGNC:18005	7q36.1
GIMAP6	GTPase, IMAP family member 6	HGNC:21918	7q36.1
GINS1	GINS complex subunit 1	HGNC:28980	20p11.21
GJC2	gap junction protein gamma 2	HGNC:17494	1q42.13
GLA	galactosidase alpha	HGNC:4296	Xq22.1

GLIS3	GLIS family zinc finger 3	HGNC:28510	9p24.2
GLRX5	glutaredoxin 5	HGNC:20134	14q32.13
GNAI2	G protein subunit alpha i2	HGNC:4385	3p21.31
GNAS	GNAS complex locus	HGNC:4392	20q13.32
GNE	glucosamine (UDP-N-acetyl)-2-epimerase/N-acetylmannosamine kinase	HGNC:23657	9p13.3
GP1BA	glycoprotein Ib platelet subunit alpha	HGNC:4439	17p13.2
GP1BB	glycoprotein Ib platelet subunit beta	HGNC:4440	22q11.21
GP6	glycoprotein VI platelet	HGNC:14388	19q13.42
GP9	glycoprotein IX platelet	HGNC:4444	3q21.3
GPC3	glypican 3	HGNC:4451	Xq26.2
GPC4	glypican 4	HGNC:4452	Xq26.2
GPR35	G protein-coupled receptor 35	HGNC:4492	2q37.3
GRIN2A	glutamate ionotropic receptor NMDA type subunit 2A	HGNC:4585	16p13.2
GSN	gelsolin	HGNC:4620	9q33.2
GTF2E2	general transcription factor IIE subunit 2	HGNC:4651	8p12
GTF2H5	general transcription factor IIH subunit 5	HGNC:21157	6q25.3
GTF3A	general transcription factor IIIA	HGNC:4662	13q12.2
GTF3AP5	general transcription factor IIIA pseudogene 5	HGNC:49748	7p21.2
GUCY2C	guanylate cyclase 2C	HGNC:4688	12p12.3
HAVCR2	hepatitis A virus cellular receptor 2	HGNC:18437	5q33.3
HAX1	HCLS1 associated protein X-1	HGNC:16915	1q21.3
HBB	hemoglobin subunit beta	HGNC:4827	11p15.4
HCK	HCK proto-oncogene, Src family tyrosine kinase	HGNC:4840	20q11.21
HELLS	helicase, lymphoid specific	HGNC:4861	10q23.33
HFE	homeostatic iron regulator	HGNC:4886	6p22.2
HIRA	histone cell cycle regulator	HGNC:4916	22q11.21
HLA-B	major histocompatibility complex, class I, B	HGNC:4932	6p21.33
HLA-DPA1	major histocompatibility complex, class II, DP alpha 1	HGNC:4938	6p21.32
HLA-DPB1	major histocompatibility complex, class II, DP beta 1	HGNC:4940	6p21.32
HLA-DQA1	major histocompatibility complex, class II, DQ alpha 1	HGNC:4942	6p21.32
HLA-DQB1	major histocompatibility complex, class II, DQ beta 1	HGNC:4944	6p21.32
HLA-DRB1	major histocompatibility complex, class II, DR beta 1	HGNC:4948	6p21.32
HMOX1	heme oxygenase 1	HGNC:5013	22q12.3
HNF1A	HNF1 homeobox A	HGNC:11621	12q24.31
HNF4A	hepatocyte nuclear factor 4 alpha	HGNC:5024	20q13.12
HNRNPK	heterogeneous nuclear ribonucleoprotein K	HGNC:5044	9q21.32
HOXA11	homeobox A11	HGNC:5101	7p15.2
HPS1	HPS1 biogenesis of lysosomal organelles complex 3 subunit 1	HGNC:5163	10q24.2
HPS3	HPS3 biogenesis of lysosomal organelles complex 2 subunit 1	HGNC:15597	3q24
HPS4	HPS4 biogenesis of lysosomal organelles complex 3 subunit 2	HGNC:15844	22q12.1
HPS5	HPS5 biogenesis of lysosomal organelles complex 2 subunit 2	HGNC:17022	11p15.1
HPS6	HPS6 biogenesis of lysosomal organelles complex 2 subunit 3	HGNC:18817	10q24.32
HR	HR lysine demethylase and nuclear receptor corepressor	HGNC:5172	8p21.3
HRAS	HRas proto-oncogene, GTPase	HGNC:5173	11p15.5
HS3ST6	heparan sulfate-glucosamine 3-sulfotransferase 6	HGNC:14178	16p13.3
HSPA1L	heat shock protein family A (Hsp70) member 1 like	HGNC:5234	6p21.33
HSPA9	heat shock protein family A (Hsp70) member 9	HGNC:5244	5q31.2
HTR1A	5-hydroxytryptamine receptor 1A	HGNC:5286	5q12.3

HTRA2	HtrA serine peptidase 2	HGNC:14348	2p13.1
HYDIN	HYDIN axonemal central pair apparatus protein	HGNC:19368	16q22.2
HYMAI	hydatidiform mole associated and imprinted	HGNC:5326	6q24.2
HYOU1	hypoxia up-regulated 1	HGNC:16931	11q23.3
ICOS	inducible T cell costimulator	HGNC:5351	2q33.2
ICOSLG	inducible T cell costimulator ligand	HGNC:17087	21q22.3
IFIH1	interferon induced with helicase C domain 1	HGNC:18873	2q24.2
IFNAR1	interferon alpha and beta receptor subunit 1	HGNC:5432	21q22.11
IFNAR2	interferon alpha and beta receptor subunit 2	HGNC:5433	21q22.11
IFNG	interferon gamma	HGNC:5438	12q15
IFNGR1	interferon gamma receptor 1	HGNC:5439	6q23.3
IFNGR2	interferon gamma receptor 2	HGNC:5440	21q22.11
IGHG1	immunoglobulin heavy constant gamma 1 (G1m marker)	HGNC:5525	14q32.33
IGHG2	immunoglobulin heavy constant gamma 2 (G2m marker)	HGNC:5526	14q32.33
IGHM	immunoglobulin heavy constant mu	HGNC:5541	14q32.33
IGKC	immunoglobulin kappa constant	HGNC:5716	2p11.2
IGLL1	immunoglobulin lambda like polypeptide 1	HGNC:5870	22q11.23
IKBKB	inhibitor of nuclear factor kappa B kinase subunit beta	HGNC:5960	8p11.21
IKBKG	inhibitor of nuclear factor kappa B kinase regulatory subunit gamma	HGNC:5961	Xq28
IKZF1	IKAROS family zinc finger 1	HGNC:13176	7p12.2
IKZF2	IKAROS family zinc finger 2	HGNC:13177	2q34
IKZF3	IKAROS family zinc finger 3	HGNC:13178	17q12-q21.1
IL10	interleukin 10	HGNC:5962	1q32.1
IL10RA	interleukin 10 receptor subunit alpha	HGNC:5964	11q23.3
IL10RB	interleukin 10 receptor subunit beta	HGNC:5965	21q22.11
IL11RA	interleukin 11 receptor subunit alpha	HGNC:5967	9p13.3
IL12A	interleukin 12A	HGNC:5969	3q25.33
IL12B	interleukin 12B	HGNC:5970	5q33.3
IL12RB1	interleukin 12 receptor subunit beta 1	HGNC:5971	19p13.11
IL12RB2	interleukin 12 receptor subunit beta 2	HGNC:5972	1p31.3
IL17A	interleukin 17A	HGNC:5981	6p12.2
IL17F	interleukin 17F	HGNC:16404	6p12.2
IL17RA	interleukin 17 receptor A	HGNC:5985	22q11.1
IL17RC	interleukin 17 receptor C	HGNC:18358	3p25.3
IL18	interleukin 18	HGNC:5986	11q23.1
IL18BP	interleukin 18 binding protein	HGNC:5987	11q13.4
IL1R1	interleukin 1 receptor type 1	HGNC:5993	2q11.2-q12.1
IL1RN	interleukin 1 receptor antagonist	HGNC:6000	2q14.1
IL21	interleukin 21	HGNC:6005	4q27
IL21R	interleukin 21 receptor	HGNC:6006	16p12.1
IL22	interleukin 22	HGNC:14900	12q15
IL23A	interleukin 23 subunit alpha	HGNC:15488	12q13.3
IL23R	interleukin 23 receptor	HGNC:19100	1p31.3
IL27RA	interleukin 27 receptor subunit alpha	HGNC:17290	19p13.12
IL2RA	interleukin 2 receptor subunit alpha	HGNC:6008	10p15.1
IL2RB	interleukin 2 receptor subunit beta	HGNC:6009	22q12.3
IL2RG	interleukin 2 receptor subunit gamma	HGNC:6010	Xq13.1
IL31RA	interleukin 31 receptor A	HGNC:18969	5q11.2

IL36RN	interleukin 36 receptor antagonist	HGNC:15561	2q14.1
IL37	interleukin 37	HGNC:15563	2q14.1
IL6	interleukin 6	HGNC:6018	7p15.3
IL6R	interleukin 6 receptor	HGNC:6019	1q21.3
IL6ST	interleukin 6 cytokine family signal transducer	HGNC:6021	5q11.2
IL7	interleukin 7	HGNC:6023	8q21.13
IL7R	interleukin 7 receptor	HGNC:6024	5p13.2
INO80	INO80 complex ATPase subunit	HGNC:26956	15q15.1
INS	insulin	HGNC:6081	11p15.5
IPO8	importin 8	HGNC:9853	12p11.21
IRAK1	interleukin 1 receptor associated kinase 1	HGNC:6112	Xq28
IRAK4	interleukin 1 receptor associated kinase 4	HGNC:17967	12q12
IRF1	interferon regulatory factor 1	HGNC:6116	5q31.1
IRF2BP2	interferon regulatory factor 2 binding protein 2	HGNC:21729	1q42.3
IRF3	interferon regulatory factor 3	HGNC:6118	19q13.33
IRF4	interferon regulatory factor 4	HGNC:6119	6p25.3
IRF5	interferon regulatory factor 5	HGNC:6120	7q32.1
IRF7	interferon regulatory factor 7	HGNC:6122	11p15.5
IRF8	interferon regulatory factor 8	HGNC:5358	16q24.1
IRF9	interferon regulatory factor 9	HGNC:6131	14q12
ISG15	ISG15 ubiquitin like modifier	HGNC:4053	1p36.33
ITCH	itchy E3 ubiquitin protein ligase	HGNC:13890	20q11.22
ITGA2	integrin subunit alpha 2	HGNC:6137	5q11.2
ITGA2B	integrin subunit alpha 2b	HGNC:6138	17q21.31
ITGA3	integrin subunit alpha 3	HGNC:6139	17q21.33
ITGAM	integrin subunit alpha M	HGNC:6149	16p11.2
ITGAV	integrin subunit alpha V	HGNC:6150	2q32.1
ITGB2	integrin subunit beta 2	HGNC:6155	21q22.3
ITGB3	integrin subunit beta 3	HGNC:6156	17q21.32
ITGB4	integrin subunit beta 4	HGNC:6158	17q25.1
ITK	IL2 inducible T cell kinase	HGNC:6171	5q33.3
ITPKB	inositol-trisphosphate 3-kinase B	HGNC:6179	1q42.12
ITPR3	inositol 1,4,5-trisphosphate receptor type 3	HGNC:6182	6p21.31
IVD	isovaleryl-CoA dehydrogenase	HGNC:6186	15q15.1
IVNS1ABP	influenza virus NS1A binding protein	HGNC:16951	1q25.3
JAGN1	jagunal vesicle mediated transporter 1	HGNC:26926	3p25.3
JAK1	Janus kinase 1	HGNC:6190	1p31.3
JAK2	Janus kinase 2	HGNC:6192	9p24.1
JAK3	Janus kinase 3	HGNC:6193	19p13.11
JAZF1	JAZF zinc finger 1	HGNC:28917	7p15.2-p15.1
JMJD1C	jumonji domain containing 1C	HGNC:12313	10q21.3
KAT6A	lysine acetyltransferase 6A	HGNC:13013	8p11.21
KCNA5	potassium voltage-gated channel subfamily A member 5	HGNC:6224	12p13.32
KCNJ11	potassium inwardly rectifying channel subfamily J member 11	HGNC:6257	11p15.1
KDM1A	lysine demethylase 1A	HGNC:29079	1p36.12
KDM6A	lysine demethylase 6A	HGNC:12637	Xp11.3
KIAA0319L	KIAA0319 like	HGNC:30071	1p34.3
KIF23	kinesin family member 23	HGNC:6392	15q23

KIT	KIT proto-oncogene, receptor tyrosine kinase	HGNC:6342	4q12
KLF1	KLF transcription factor 1	HGNC:6345	19p13.13
KLF11	KLF transcription factor 11	HGNC:11811	2p25.1
KLHDC8B	kelch domain containing 8B	HGNC:28557	3p21.31
KLLN	killin, p53 regulated DNA replication inhibitor	HGNC:37212	10q23
KMT2A	lysine methyltransferase 2A	HGNC:7132	11q23.3
KMT2D	lysine methyltransferase 2D	HGNC:7133	12q13.12
KNG1	kininogen 1	HGNC:6383	3q27.3
KNSTRN	kinetochore localized astrin (SPAG5) binding protein	HGNC:30767	15q15.1
KRAS	KRAS proto-oncogene, GTPase	HGNC:6407	12p12.1
KRT14	keratin 14	HGNC:6416	17q21.2
KRT5	keratin 5	HGNC:6442	12q13.13
KRT74	keratin 74	HGNC:28929	12q13.13
KRT9	keratin 9	HGNC:6447	17q21.2
LACC1	laccase domain containing 1	HGNC:26789	13q14.11
LAMA3	laminin subunit alpha 3	HGNC:6483	18q11.2
LAMB3	laminin subunit beta 3	HGNC:6490	1q32.2
LAMC2	laminin subunit gamma 2	HGNC:6493	1q25.3
LAMTOR2	late endosomal/lysosomal adaptor, MAPK and MTOR activator 2	HGNC:29796	1q22
LARS2	leucyl-tRNA synthetase 2, mitochondrial	HGNC:17095	3p21.31
LAT	linker for activation of T cells	HGNC:18874	16p11.2
LBR	lamin B receptor	HGNC:6518	1q42.12
LCK	LCK proto-oncogene, Src family tyrosine kinase	HGNC:6524	1p35.2
LCP2	lymphocyte cytosolic protein 2	HGNC:6529	5q35.1
LCT	lactase	HGNC:6530	2q21.3
LEMD3	LEM domain containing 3	HGNC:28887	12q14.3
LETM1	leucine zipper and EF-hand containing transmembrane protein 1	HGNC:6556	4p16.3
LIG1	DNA ligase 1	HGNC:6598	19q13.33
LIG4	DNA ligase 4	HGNC:6601	13q33.3
LIPA	lipase A, lysosomal acid type	HGNC:6617	10q23.31
LMBRD1	LMBR1 domain containing 1	HGNC:23038	6q13
LMNB2	lamin B2	HGNC:6638	19p13.3
LPIN2	lipin 2	HGNC:14450	18p11.31
LRBA	LPS responsive beige-like anchor protein	HGNC:1742	4q31.3
LRP5	LDL receptor related protein 5	HGNC:6697	11q13.2
LRRC32	leucine rich repeat containing 32	HGNC:4161	11q13.5
LRRC56	leucine rich repeat containing 56	HGNC:25430	11p15.5
LRRC8A	leucine rich repeat containing 8 VRAC subunit A	HGNC:19027	9q34.11
LSM11	LSM11, U7 small nuclear RNA associated	HGNC:30860	5q33.3
LYN	LYN proto-oncogene, Src family tyrosine kinase	HGNC:6735	8q12.1
LYST	lysosomal trafficking regulator	HGNC:1968	1q42.3
LYZ	lysozyme	HGNC:6740	12q15
LZTR1	leucine zipper like post translational regulator 1	HGNC:6742	22q11.21
MAD2L2	mitotic arrest deficient 2 like 2	HGNC:6764	1p36.22
MAGT1	magnesium transporter 1	HGNC:28880	Xq21.1
MALT1	MALT1 paracaspase	HGNC:6819	18q21.32
MAN2B1	mannosidase alpha class 2B member 1	HGNC:6826	19p13.13
MAN2B2	mannosidase alpha class 2B member 2	HGNC:29623	4p16.1

MANBA	mannosidase beta	HGNC:6831	4q24
MAP1LC3B2	microtubule associated protein 1 light chain 3 beta 2	HGNC:34390	12q24.22
MAP2K1	mitogen-activated protein kinase kinase 1	HGNC:6840	15q22.31
MAP2K2	mitogen-activated protein kinase kinase 2	HGNC:6842	19p13.3
MAP3K14	mitogen-activated protein kinase kinase kinase 14	HGNC:6853	17q21.31
MAP3K8	mitogen-activated protein kinase kinase kinase 8	HGNC:6860	10p11.23
MAPK1	mitogen-activated protein kinase 1	HGNC:6871	22q11.22
MAPK8	mitogen-activated protein kinase 8	HGNC:6881	10q11.22
MASP1	MBL associated serine protease 1	HGNC:6901	3q27.3
MASP2	MBL associated serine protease 2	HGNC:6902	1p36.22
MASTL	microtubule associated serine/threonine kinase like	HGNC:19042	10p12.1
MAT2A	methionine adenosyltransferase 2A	HGNC:6904	2p11.2
MBD4	methyl-CpG binding domain 4, DNA glycosylase	HGNC:6919	3q21.3
MBL2	mannose binding lectin 2	HGNC:6922	10q21.1
MBTPS2	membrane bound transcription factor peptidase, site 2	HGNC:15455	Xp22.12
MC2R	melanocortin 2 receptor	HGNC:6930	18p11.21
MCIDAS	multiciliate differentiation and DNA synthesis associated cell cycle protein	HGNC:40050	5q11.2
MCM10	minichromosome maintenance 10 replication initiation factor	HGNC:18043	10p13
MCM4	minichromosome maintenance complex component 4	HGNC:6947	8q11.21
MCTS1	MCTS1 re-initiation and release factor	HGNC:23357	Xq24
MECOM	MDS1 and EVI1 complex locus	HGNC:3498	3q26.2
MECP2	methyl-CpG binding protein 2	HGNC:6990	Xq28
MED13L	mediator complex subunit 13L	HGNC:22962	12q24.21
MEFV	MEFV innate immunity regulator, pyrin	HGNC:6998	16p13.3
MEIS2	Meis homeobox 2	HGNC:7001	15q14
MGAT2	alpha-1,6-mannosyl-glycoprotein 2-beta-N-acetylglucosaminyltransferase	HGNC:7045	14q21.3
MICA	MHC class I polypeptide-related sequence A	HGNC:7090	6p21.33
MIF	macrophage migration inhibitory factor	HGNC:7097	22q11.23
MLH1	mutL homolog 1	HGNC:7127	3p22.2
MLPH	melanophilin	HGNC:29643	2q37.3
MMAA	metabolism of cobalamin associated A	HGNC:18871	4q31.21
MMAB	metabolism of cobalamin associated B	HGNC:19331	12q24.11
MMACHC	metabolism of cobalamin associated C	HGNC:24525	1p34.1
MMADHC	metabolism of cobalamin associated D	HGNC:25221	2q23.2
MMEL1	membrane metalloendopeptidase like 1	HGNC:14668	1p36.32
MMP2	matrix metallopeptidase 2	HGNC:7166	16q12.2
MMUT	methylmalonyl-CoA mutase	HGNC:7526	6p12.3
MOGS	mannosyl-oligosaccharide glucosidase	HGNC:24862	2p13.1
MPEG1	macrophage expressed 1	HGNC:29619	11q12.1
MPI	mannose phosphate isomerase	HGNC:7216	15q24.1
MPL	MPL proto-oncogene, thrombopoietin receptor	HGNC:7217	1p34.2
MPLKIP	M-phase specific PLK1 interacting protein	HGNC:16002	7p14.1
MPO	myeloperoxidase	HGNC:7218	17q22
MR1	major histocompatibility complex, class I-related	HGNC:4975	1q25.3
MRAP	melanocortin 2 receptor accessory protein	HGNC:1304	21q22.11
MRAS	muscle RAS oncogene homolog	HGNC:7227	3q22.3
MRE11	MRE11 double strand break repair nuclease	HGNC:7230	11q21

MRTFA	myocardin related transcription factor A	HGNC:14334	22q13.1-q13.2
MS4A1	membrane spanning 4-domains A1	HGNC:7315	11q12.2
MSH2	mutS homolog 2	HGNC:7325	2p21-p16.3
MSH6	mutS homolog 6	HGNC:7329	2p16.3
MSN	moesin	HGNC:7373	Xq12
MST1	macrophage stimulating 1	HGNC:7380	3p21.31
MTHFD1	methylenetetrahydrofolate dehydrogenase, cyclohydrolase and formyltetrahydrofolate synthetase 1	HGNC:7432	14q23.3
MTOR	mechanistic target of rapamycin kinase	HGNC:3942	1p36.22
MTPAP	mitochondrial poly(A) polymerase	HGNC:25532	10p11.23
MTRR	5-methyltetrahydrofolate-homocysteine methyltransferase reductase	HGNC:7473	5p15.31
MUC5B	mucin 5B, oligomeric mucus/gel-forming	HGNC:7516	11p15.5
MVK	mevalonate kinase	HGNC:7530	12q24.11
MYC	MYC proto-oncogene, bHLH transcription factor	HGNC:7553	8q24.21
MYD88	MYD88 innate immune signal transduction adaptor	HGNC:7562	3p22.2
MYH9	myosin heavy chain 9	HGNC:7579	22q12.3
MYO5A	myosin VA	HGNC:7602	15q21.2
MYO5B	myosin VB	HGNC:7603	18q21.1
MYO9A	myosin IXA	HGNC:7608	15q23
MYOF	myoferlin	HGNC:3656	10q23.33
MYSM1	Myb like, SWIRM and MPN domains 1	HGNC:29401	1p32.1
NAE1	NEDD8 activating enzyme E1 subunit 1	HGNC:621	16q22.1
NAF1	nuclear assembly factor 1 ribonucleoprotein	HGNC:25126	4q32.2
NARS2	asparaginyl-tRNA synthetase 2, mitochondrial	HGNC:26274	11q14.1
NBAS	NBAS subunit of NRZ tethering complex	HGNC:15625	2p24.3
NBEAL2	neurobeachin like 2	HGNC:31928	3p21.31
NBN	nibrin	HGNC:7652	8q21.3
NCF1	neutrophil cytosolic factor 1	HGNC:7660	7q11.23
NCF2	neutrophil cytosolic factor 2	HGNC:7661	1q25.3
NCF4	neutrophil cytosolic factor 4	HGNC:7662	22q12.3
NCKAP1L	NCK associated protein 1 like	HGNC:4862	12q13.13-q13.2
NCSTN	nicastrin	HGNC:17091	1q23.2
NDUFB11	NADH:ubiquinone oxidoreductase subunit B11	HGNC:20372	Xp11.3
NEUROD1	neuronal differentiation 1	HGNC:7762	2q31.3
NEUROG3	neurogenin 3	HGNC:13806	10q22.1
NF1	neurofibromin 1	HGNC:7765	17q11.2
NF2	NF2, moesin-ezrin-radixin like (MERLIN) tumor suppressor	HGNC:7773	22q12.2
NFAT5	nuclear factor of activated T cells 5	HGNC:7774	16q22.1
NFE2L2	NFE2 like bZIP transcription factor 2	HGNC:7782	2q31.2
NFIL3	nuclear factor, interleukin 3 regulated	HGNC:7787	9q22.31
NFKB1	nuclear factor kappa B subunit 1	HGNC:7794	4q24
NFKB2	nuclear factor kappa B subunit 2	HGNC:7795	10q24.32
NFKBIA	NFKB inhibitor alpha	HGNC:7797	14q13.2
NFKBID	NFKB inhibitor delta	HGNC:15671	19q13.12
NFKBIL1	NFKB inhibitor like 1	HGNC:7800	6p21.33
NHEJ1	non-homologous end joining factor 1	HGNC:25737	2q35
NHLRC2	NHL repeat containing 2	HGNC:24731	10q25.3
NHP2	NHP2 ribonucleoprotein	HGNC:14377	5q35.3
NLRC4	NLR family CARD domain containing 4	HGNC:16412	2p22.3

NLRP1	NLR family pyrin domain containing 1	HGNC:14374	17p13
NLRP12	NLR family pyrin domain containing 12	HGNC:22938	19q13.42
NLRP3	NLR family pyrin domain containing 3	HGNC:16400	1q44
NLRP6	NLR family pyrin domain containing 6	HGNC:22944	11p15.5
NME8	NME/NM23 family member 8	HGNC:16473	7p14.1
NNT	nicotinamide nucleotide transhydrogenase	HGNC:7863	5p12
NOD2	nucleotide binding oligomerization domain containing 2	HGNC:5331	16q12.1
NOP10	NOP10 ribonucleoprotein	HGNC:14378	15q14
NOS2	nitric oxide synthase 2	HGNC:7873	17q11.2
NOTCH2	notch receptor 2	HGNC:7882	1p12
NPAT	nuclear protein, coactivator of histone transcription	HGNC:7896	11q22.3
NPC1	NPC intracellular cholesterol transporter 1	HGNC:7897	18q11.2
NPM1	nucleophosmin 1	HGNC:7910	5q35.1
NR1H4	nuclear receptor subfamily 1 group H member 4	HGNC:7967	12q23.1
NR3C1	nuclear receptor subfamily 3 group C member 1	HGNC:7978	5q31.3
NRAS	NRAS proto-oncogene, GTPase	HGNC:7989	1p13.2
NSD2	nuclear receptor binding SET domain protein 2	HGNC:12766	4p16.3
NSMCE2	NSE2 SUMO ligase component of SMC5/6 complex	HGNC:26513	8q24.13
NSMCE3	NSE3 component of SMC5/6 complex	HGNC:7677	15q13.1
NUDCD3	NudC domain containing 3	HGNC:22208	7p13
NUP214	nucleoporin 214	HGNC:8064	9q34.13
OAS1	2'-5'-oligoadenylate synthetase 1	HGNC:8086	12q24.13
ODC1	ornithine decarboxylase 1	HGNC:8109	2p25.1
OFD1	OFD1 centriole and centriolar satellite protein	HGNC:2567	Xp22.2
OGFRL1	opioid growth factor receptor like 1	HGNC:21378	6q13
ORAI1	ORAI calcium release-activated calcium modulator 1	HGNC:25896	12q24.31
OSMR	oncostatin M receptor	HGNC:8507	5p13.1
OSTM1	osteoclastogenesis associated transmembrane protein 1	HGNC:21652	6q21
OTUD6B	OTU deubiquitinase 6B	HGNC:24281	8q21.3
OTULIN	OTU deubiquitinase with linear linkage specificity	HGNC:25118	5p15.2
P2RY12	purinergic receptor P2Y12	HGNC:18124	3q25.1
PALB2	partner and localizer of BRCA2	HGNC:26144	16p12.2
PARN	poly(A)-specific ribonuclease	HGNC:8609	16p13.12
PARP1	poly(ADP-ribose) polymerase 1	HGNC:270	1q42.12
PAX1	paired box 1	HGNC:8615	20p11.22
PAX4	paired box 4	HGNC:8618	7q32.1
PAX5	paired box 5	HGNC:8619	9p13.2
PCCA	propionyl-CoA carboxylase subunit alpha	HGNC:8653	13q32.3
PCCB	propionyl-CoA carboxylase subunit beta	HGNC:8654	3q22.3
PDCD1	programmed cell death 1	HGNC:8760	2q37.3
PDX1	pancreatic and duodenal homeobox 1	HGNC:6107	13q12.2
PEPD	peptidase D	HGNC:8840	19q13.11
PGM3	phosphoglucomutase 3	HGNC:8907	6q14.1
PI4KA	phosphatidylinositol 4-kinase alpha	HGNC:8983	22q11.21
PIGA	phosphatidylinositol glycan anchor biosynthesis class A	HGNC:8957	Xp22.2
PIGT	phosphatidylinositol glycan anchor biosynthesis class T	HGNC:14938	20q13.12
PIK3CA	phosphatidylinositol-4,5-bisphosphate 3-kinase catalytic subunit alpha	HGNC:8975	3q26.32

PIK3CD	phosphatidylinositol-4,5-bisphosphate 3-kinase catalytic subunit delta	HGNC:8977	1p36.22
PIK3CG	phosphatidylinositol-4,5-bisphosphate 3-kinase catalytic subunit gamma	HGNC:8978	7q22.3
PIK3R1	phosphoinositide-3-kinase regulatory subunit 1	HGNC:8979	5q13.1
PLA2G4A	phospholipase A2 group IVA	HGNC:9035	1q31.1
PLAGL1	PLAG1 like zinc finger 1	HGNC:9046	6q24.2
PLCG1	phospholipase C gamma 1	HGNC:9065	20q12
PLCG2	phospholipase C gamma 2	HGNC:9066	16q24.1
PLEC	plectin	HGNC:9069	8q24.3
PLEKHM1	pleckstrin homology and RUN domain containing M1	HGNC:29017	17q21.31
PLG	plasminogen	HGNC:9071	6q26
PLVAP	plasmalemma vesicle associated protein	HGNC:13635	19p13.11
PMM2	phosphomannomutase 2	HGNC:9115	16p13.2
PMS2	PMS1 homolog 2, mismatch repair system component	HGNC:9122	7p22.1
PNLIP	pancreatic lipase	HGNC:9155	10q25.3
PNP	purine nucleoside phosphorylase	HGNC:7892	14q11.2
POGLUT1	protein O-glucosyltransferase 1	HGNC:22954	3q13.33
POGZ	pogo transposable element derived with ZNF domain	HGNC:18801	1q21.3
POLA1	DNA polymerase alpha 1, catalytic subunit	HGNC:9173	Xp22.11-p21.3
POLD1	DNA polymerase delta 1, catalytic subunit	HGNC:9175	19q13.33
POLD2	DNA polymerase delta 2, accessory subunit	HGNC:9176	7p13
POLD3	DNA polymerase delta 3, accessory subunit	HGNC:20932	11q13.4
POLE	DNA polymerase epsilon, catalytic subunit	HGNC:9177	12q24.33
POLE2	DNA polymerase epsilon 2, accessory subunit	HGNC:9178	14q21.3
POLG	DNA polymerase gamma, catalytic subunit	HGNC:9179	15q26.1
POLR3A	RNA polymerase III subunit A	HGNC:30074	10q22.3
POLR3C	RNA polymerase III subunit C	HGNC:30076	1q21.1
POLR3F	RNA polymerase III subunit F	HGNC:15763	20p11.23
POMP	proteasome maturation protein	HGNC:20330	13q12.3
POT1	protection of telomeres 1	HGNC:17284	7q31.33
POU2AF1	POU class 2 homeobox associating factor 1	HGNC:9211	11q23.1
PPP1CB	protein phosphatase 1 catalytic subunit beta	HGNC:9282	2p23.2
PRDX1	peroxiredoxin 1	HGNC:9352	1p34.1
PREPL	prolyl endopeptidase like	HGNC:30228	2p21
PRF1	perforin 1	HGNC:9360	10q22.1
PRG4	proteoglycan 4	HGNC:9364	1q31.1
PRIM1	DNA primase subunit 1	HGNC:9369	12q13.3
PRKACG	protein kinase cAMP-activated catalytic subunit gamma	HGNC:9382	9q21.11
PRKCD	protein kinase C delta	HGNC:9399	3p21.1
PRKDC	protein kinase, DNA-activated, catalytic subunit	HGNC:9413	8q11.21
PRPS1	phosphoribosyl pyrophosphate synthetase 1	HGNC:9462	Xq22.3
PRTN3	proteinase 3	HGNC:9495	19p13.3
PSEN1	presenilin 1	HGNC:9508	14q24.2
PSENEN	presenilin enhancer, gamma-secretase subunit	HGNC:30100	19q13.12
PSMA3	proteasome 20S subunit alpha 3	HGNC:9532	14q23.1
PSMA5	proteasome 20S subunit alpha 5	HGNC:9534	1p13.3
PSMB10	proteasome 20S subunit beta 10	HGNC:9538	16q22.1
PSMB4	proteasome 20S subunit beta 4	HGNC:9541	1q21.3

PSMB8	proteasome 20S subunit beta 8	HGNC:9545	6p21.32
PSMB9	proteasome 20S subunit beta 9	HGNC:9546	6p21.32
PSMC5	proteasome 26S subunit, ATPase 5	HGNC:9552	17q23.3
PSMG2	proteasome assembly chaperone 2	HGNC:24929	18p11.21
PSTPIP1	proline-serine-threonine phosphatase interacting protein 1	HGNC:9580	15q24.3
PTCRA	pre T cell antigen receptor alpha	HGNC:21290	6p21.1
PTEN	phosphatase and tensin homolog	HGNC:9588	10q23.31
PTPN11	protein tyrosine phosphatase non-receptor type 11	HGNC:9644	12q24.13
PTPN2	protein tyrosine phosphatase non-receptor type 2	HGNC:9650	18p11.21
PTPN22	protein tyrosine phosphatase non-receptor type 22	HGNC:9652	1p13.2
PTPRC	protein tyrosine phosphatase receptor type C	HGNC:9666	1q31.3-q32.1
PTX3	pentraxin 3	HGNC:9692	3q25.32
PUS1	pseudouridine synthase 1	HGNC:15508	12q24.33
PXK	PX domain containing serine/threonine kinase like	HGNC:23326	3p14.3
QSOX2	quiescin sulfhydryl oxidase 2	HGNC:30249	9q34.3
RAB27A	RAB27A, member RAS oncogene family	HGNC:9766	15q21.3
RAC2	Rac family small GTPase 2	HGNC:9802	22q13.1
RAD50	RAD50 double strand break repair protein	HGNC:9816	5q31.1
RAD51	RAD51 recombinase	HGNC:9817	15q15.1
RAD51C	RAD51 paralog C	HGNC:9820	17q22
RAF1	Raf-1 proto-oncogene, serine/threonine kinase	HGNC:9829	3p25.2
RAG1	recombination activating 1	HGNC:9831	11p12
RAG2	recombination activating 2	HGNC:9832	11p12
RANBP2	RAN binding protein 2	HGNC:9848	2q13
RAP1A	RAP1A, member of RAS oncogene family	HGNC:9855	1p13.2
RAP1B	RAP1B, member of RAS oncogene family	HGNC:9857	12q15
RAPSN	receptor associated protein of the synapse	HGNC:9863	11p11.2
RASA2	RAS p21 protein activator 2	HGNC:9872	3q23
RASGRP1	RAS guanyl releasing protein 1	HGNC:9878	15q14
RASGRP2	RAS guanyl releasing protein 2	HGNC:9879	11q13.1
RBCK1	RANBP2-type and C3HC4-type zinc finger containing 1	HGNC:15864	20p13
RBM8A	RNA binding motif protein 8A	HGNC:9905	1q21.1
RC3H1	ring finger and CCCH-type domains 1	HGNC:29434	1q25.1
RECQL4	RecQ like helicase 4	HGNC:9949	8q24.3
REL	REL proto-oncogene, NF-kB subunit	HGNC:9954	2p16.1
RELA	RELA proto-oncogene, NF-kB subunit	HGNC:9955	11q13.1
RELB	RELB proto-oncogene, NF-kB subunit	HGNC:9956	19q13.32
RELN	reelin	HGNC:9957	7q22.1
RET	ret proto-oncogene	HGNC:9967	10q11.21
REXO2	RNA exonuclease 2	HGNC:17851	11q23.2
RFWD3	ring finger and WD repeat domain 3	HGNC:25539	16q23.1
RFX5	regulatory factor X5	HGNC:9986	1q21.3
RFXANK	regulatory factor X associated ankyrin containing protein	HGNC:9987	19p13.11
RFXAP	regulatory factor X associated protein	HGNC:9988	13q13.3
RGS10	regulator of G protein signaling 10	HGNC:9992	10q26.11
RHAG	Rh associated glycoprotein	HGNC:10006	6p12.3
RHBDF2	rhomboid 5 homolog 2	HGNC:20788	17q25.1
RHCE	Rh blood group CcEe antigens	HGNC:10008	1p36.11

RHD	Rh blood group D antigen	HGNC:10009	1p36.11
RHOG	ras homolog family member G	HGNC:672	11p15.4
RHOH	ras homolog family member H	HGNC:686	4p14
RIPK1	receptor interacting serine/threonine kinase 1	HGNC:10019	6p25.2
RIT1	Ras like without CAAX 1	HGNC:10023	1q22
RMRP	RNA component of mitochondrial RNA processing endoribonuclease	HGNC:10031	9p13.3
RNASEH2A	ribonuclease H2 subunit A	HGNC:18518	19p13.13
RNASEH2B	ribonuclease H2 subunit B	HGNC:25671	13q14.3
RNASEH2C	ribonuclease H2 subunit C	HGNC:24116	11q13.1
RNF113A	ring finger protein 113A	HGNC:12974	Xq24
RNF168	ring finger protein 168	HGNC:26661	3q29
RNF31	ring finger protein 31	HGNC:16031	14q12
RNU4ATAC	RNA, U4atac small nuclear	HGNC:34016	2q14.2
RNU7-1	RNA, U7 small nuclear 1	HGNC:34033	12p13.31
RORC	RAR related orphan receptor C	HGNC:10260	1q21.3
RPA1	replication protein A1	HGNC:10289	17p13.3
RPGR	retinitis pigmentosa GTPase regulator	HGNC:10295	Xp11.4
RPL10	ribosomal protein L10	HGNC:10298	Xq28
RPL11	ribosomal protein L11	HGNC:10301	1p36.11
RPL15	ribosomal protein L15	HGNC:10306	3p24.2
RPL18	ribosomal protein L18	HGNC:10310	19q13.33
RPL19	ribosomal protein L19	HGNC:10312	17q12
RPL23	ribosomal protein L23	HGNC:10316	17q12
RPL26	ribosomal protein L26	HGNC:10327	17p13.1
RPL27	ribosomal protein L27	HGNC:10328	17q21.31
RPL31	ribosomal protein L31	HGNC:10334	2q11.2
RPL35	ribosomal protein L35	HGNC:10344	9q33.3
RPL35A	ribosomal protein L35a	HGNC:10345	3q29
RPL36	ribosomal protein L36	HGNC:13631	19p13.3
RPL5	ribosomal protein L5	HGNC:10360	1p22.1
RPL9	ribosomal protein L9	HGNC:10369	4p14
RPS14	ribosomal protein S14	HGNC:10387	5q33.1
RPS15	ribosomal protein S15	HGNC:10388	19p13.3
RPS15A	ribosomal protein S15a	HGNC:10389	16p12.3
RPS19	ribosomal protein S19	HGNC:10402	19q13.2
RPS24	ribosomal protein S24	HGNC:10411	10q22.3
RPS26	ribosomal protein S26	HGNC:10414	12q13.2
RPS27	ribosomal protein S27	HGNC:10416	1q21.3
RPS27A	ribosomal protein S27a	HGNC:10417	2p16.1
RPS28	ribosomal protein S28	HGNC:10418	19p13.2
RPS29	ribosomal protein S29	HGNC:10419	14q21.3
RPS7	ribosomal protein S7	HGNC:10440	2p25.3
RPSA	ribosomal protein SA	HGNC:6502	3p22.1
RRAS	RAS related	HGNC:10447	19q13.33
RRAS2	RAS related 2	HGNC:17271	11p15.2
RREB1	ras responsive element binding protein 1	HGNC:10449	6p24.3
RSPH1	radial spoke head component 1	HGNC:12371	21q22.3
RSPH3	radial spoke head 3	HGNC:21054	6q25.3

RSPH4A	radial spoke head component 4A	HGNC:21558	6q22.1
RSPH9	radial spoke head component 9	HGNC:21057	6p21.1
RTEL1	regulator of telomere elongation helicase 1	HGNC:15888	20q13.33
RUNX1	RUNX family transcription factor 1	HGNC:10471	21q22.12
SAMD3	sterile alpha motif domain containing 3	HGNC:21574	6q23.1
SAMD9	sterile alpha motif domain containing 9	HGNC:1348	7q21.2
SAMD9L	sterile alpha motif domain containing 9 like	HGNC:1349	7q21.2
SAMHD1	SAM and HD domain containing deoxynucleoside triphosphate triphosphohydrolase 1	HGNC:15925	20q11.23
SAR1B	secretion associated Ras related GTPase 1B	HGNC:10535	5q31.1
SART3	spliceosome associated factor 3, U4/U6 recycling protein	HGNC:16860	12q23.3
SASH3	SAM and SH3 domain containing 3	HGNC:15975	Xq26.1
SAT1	spermidine/spermine N1-acetyltransferase 1	HGNC:10540	Xp22.11
SBDS	SBDS ribosome maturation factor	HGNC:19440	7q11.21
SBF2	SET binding factor 2	HGNC:2135	11p15.4
SC5D	sterol-C5-desaturase	HGNC:10547	11q23.3-q24.1
SCARB2	scavenger receptor class B member 2	HGNC:1665	4q21.1
SCGN	secretagogin, EF-hand calcium binding protein	HGNC:16941	6p22.2
SCN4A	sodium voltage-gated channel alpha subunit 4	HGNC:10591	17q23.3
SCO2	synthesis of cytochrome C oxidase 2	HGNC:10604	22q13.33
SDHB	succinate dehydrogenase complex iron sulfur subunit B	HGNC:10681	1p36.13
SDHC	succinate dehydrogenase complex subunit C	HGNC:10682	1q23.3
SDHD	succinate dehydrogenase complex subunit D	HGNC:10683	11q23.1
SEC23B	SEC23 homolog B, COPII component	HGNC:10702	20p11.23
SEC24C	SEC24 homolog C, COPII component	HGNC:10705	10q22.2
SEC61A1	SEC61 translocon subunit alpha 1	HGNC:18276	3q21.3
SEMA3E	semaphorin 3E	HGNC:10727	7q21.11
SEMA4D	semaphorin 4D	HGNC:10732	9q22.2
SEMA6B	semaphorin 6B	HGNC:10739	19p13.3
SERPINA1	serpin family A member 1	HGNC:8941	14q32.13
SERPINB1	serpin family B member 1	HGNC:3311	6p25.2
SERPING1	serpin family G member 1	HGNC:1228	11q12.1
SETX	senataxin	HGNC:445	9q34.13
SFTPA2	surfactant protein A2	HGNC:10799	10q22.3
SFTPC	surfactant protein C	HGNC:10802	8p21.3
SGPL1	sphingosine-1-phosphate lyase 1	HGNC:10817	10q22.1
SH2D1A	SH2 domain containing 1A	HGNC:10820	Xq25
SH3BP2	SH3 domain binding protein 2	HGNC:10825	4p16.3
SH3KBP1	SH3 domain containing kinase binding protein 1	HGNC:13867	Xp22.12
SHANK3	SH3 and multiple ankyrin repeat domains 3	HGNC:14294	22q13.33
SHARPIN	SHANK associated RH domain interactor	HGNC:25321	8q24.3
SHOC2	SHOC2 leucine rich repeat scaffold protein	HGNC:15454	10q25.2
SI	sucrase-isomaltase	HGNC:10856	3q26.1
SIAE	sialic acid acetylesterase	HGNC:18187	11q24.2
SIK3	SIK family kinase 3	HGNC:29165	11q23.3
SIRT1	sirtuin 1	HGNC:14929	10q21.3
SKIV2L	SKI2 subunit of superkiller complex	HGNC:10898	6p21.33
SLC10A2	solute carrier family 10 member 2	HGNC:10906	13q33.1
SLC12A3	solute carrier family 12 member 3	HGNC:10912	16q13

SLC13A4	solute carrier family 13 member 4	HGNC:15827	7q33
SLC18A3	solute carrier family 18 member A3	HGNC:10936	10q11.23
SLC19A1	solute carrier family 19 member 1	HGNC:10937	21q22.3
SLC19A2	solute carrier family 19 member 2	HGNC:10938	1q24.2
SLC22A4	solute carrier family 22 member 4	HGNC:10968	5q31.1
SLC25A1	solute carrier family 25 member 1	HGNC:10979	22q11.21
SLC25A13	solute carrier family 25 member 13	HGNC:10983	7q21.3
SLC25A38	solute carrier family 25 member 38	HGNC:26054	3p22.1
SLC26A3	solute carrier family 26 member 3	HGNC:3018	7q22.3-q31.1
SLC29A3	solute carrier family 29 member 3	HGNC:23096	10q22.1
SLC35A1	solute carrier family 35 member A1	HGNC:11021	6q15
SLC35C1	solute carrier family 35 member C1	HGNC:20197	11p11.2
SLC37A4	solute carrier family 37 member 4	HGNC:4061	11q23.3
SLC39A4	solute carrier family 39 member 4	HGNC:17129	8q24.3
SLC39A7	solute carrier family 39 member 7	HGNC:4927	6p21.32
SLC39A8	solute carrier family 39 member 8	HGNC:20862	4q24
SLC46A1	solute carrier family 46 member 1	HGNC:30521	17q11.2
SLC51B	SLC51 subunit beta	HGNC:29956	15q22.31
SLC5A1	solute carrier family 5 member 1	HGNC:11036	22q12.3
SLC5A6	solute carrier family 5 member 6	HGNC:11041	2p23.3
SLC5A7	solute carrier family 5 member 7	HGNC:14025	2q12.3
SLC7A7	solute carrier family 7 member 7	HGNC:11065	14q11.2
SLC9A3	solute carrier family 9 member A3	HGNC:11073	5p15.33
SLCO2A1	solute carrier organic anion transporter family member 2A1	HGNC:10955	3q22.1-q22.2
SLFN14	schlafen family member 14	HGNC:32689	17q12
SLX4	SLX4 structure-specific endonuclease subunit	HGNC:23845	16p13.3
SMAD2	SMAD family member 2	HGNC:6768	18q21.1
SMARCAL1	SNF2 related chromatin remodeling annealing helicase 1	HGNC:11102	2q35
SMARCD2	SWI/SNF related BAF chromatin remodeling complex subunit D2	HGNC:11107	17q23.3
SMC1A	structural maintenance of chromosomes 1A	HGNC:11111	Xp11.22
SMC3	structural maintenance of chromosomes 3	HGNC:2468	10q25.2
SMPD1	sphingomyelin phosphodiesterase 1	HGNC:11120	11p15.4
SNAP25	synaptosome associated protein 25	HGNC:11132	20p12.2
SNORA31	small nucleolar RNA, H/ACA box 31	HGNC:32621	13q14.13
SNX10	sorting nexin 10	HGNC:14974	7p15.2
SOCS1	suppressor of cytokine signaling 1	HGNC:19383	16p13.13
SON	SON DNA and RNA binding protein	HGNC:11183	21q22.11
SOS1	SOS Ras/Rac guanine nucleotide exchange factor 1	HGNC:11187	2p22.1
SOS2	SOS Ras/Rho guanine nucleotide exchange factor 2	HGNC:11188	14q21.3
SP1	Sp1 transcription factor	HGNC:11205	12q13.13
SP110	SP110 nuclear body protein	HGNC:5401	2q37.1
SPAG1	sperm associated antigen 1	HGNC:11212	8q22.2
SPATA5	AAA ATPase AFG2A	HGNC:18119	4q28.1
SPI1	Spi-1 proto-oncogene	HGNC:11241	11p11.2
SPIB	Spi-B transcription factor	HGNC:11242	19q13.33
SPINK5	serine peptidase inhibitor Kazal type 5	HGNC:15464	5q32
SPINT2	serine peptidase inhibitor, Kunitz type 2	HGNC:11247	19q13.2
SPP1	secreted phosphoprotein 1	HGNC:11255	4q22.1

SPPL2A	signal peptide peptidase like 2A	HGNC:30227	15q21.2
SPRED1	sprouty related EVH1 domain containing 1	HGNC:20249	15q14
SRC	SRC proto-oncogene, non-receptor tyrosine kinase	HGNC:11283	20q11.23
SRP19	signal recognition particle 19	HGNC:11300	5q22.2
SRP54	signal recognition particle 54	HGNC:11301	14q13.2
SRP72	signal recognition particle 72	HGNC:11303	4q12
STAR	steroidogenic acute regulatory protein	HGNC:11359	8p11.23
STAT1	signal transducer and activator of transcription 1	HGNC:11362	2q32.2
STAT2	signal transducer and activator of transcription 2	HGNC:11363	12q13.3
STAT3	signal transducer and activator of transcription 3	HGNC:11364	17q21.2
STAT4	signal transducer and activator of transcription 4	HGNC:11365	2q32.2-q32.3
STAT5A	signal transducer and activator of transcription 5A	HGNC:11366	17q21.2
STAT5B	signal transducer and activator of transcription 5B	HGNC:11367	17q21.2
STAT6	signal transducer and activator of transcription 6	HGNC:11368	12q13.3
STIM1	stromal interaction molecule 1	HGNC:11386	11p15.4
STING1	stimulator of interferon response cGAMP interactor 1	HGNC:27962	5q31.2
STK36	serine/threonine kinase 36	HGNC:17209	2q35
STK4	serine/threonine kinase 4	HGNC:11408	20q13.12
STN1	STN1 subunit of CST complex	HGNC:26200	10q24.33
STOX1	storkhead box 1	HGNC:23508	10q22.1
STX11	syntaxin 11	HGNC:11429	6q24.2
STX16	syntaxin 16	HGNC:11431	20q13.32
STX3	syntaxin 3	HGNC:11438	11q12.1
STXBP2	syntaxin binding protein 2	HGNC:11445	19p13.2
STXBP3	syntaxin binding protein 3	HGNC:11446	1p13.3
SYK	spleen associated tyrosine kinase	HGNC:11491	9q22.2
SYT2	synaptotagmin 2	HGNC:11510	1q32.1
TAFAZZIN	tafazzin, phospholipid-lysophospholipid transacylase	HGNC:11577	Xq28
TAOK2	TAO kinase 2	HGNC:16835	16p11.2
TAP1	transporter 1, ATP binding cassette subfamily B member	HGNC:43	6p21.32
TAP2	transporter 2, ATP binding cassette subfamily B member	HGNC:44	6p21.32
TAPBP	TAP binding protein	HGNC:11566	6p21.32
TARS1	threonyl-tRNA synthetase 1	HGNC:11572	5p13.3
TASP1	taspase 1	HGNC:15859	20p12.1
TBCE	tubulin folding cofactor E	HGNC:11582	1q42.3
TBK1	TANK binding kinase 1	HGNC:11584	12q14.2
TBX1	T-box transcription factor 1	HGNC:11592	22q11.21
TBX2	T-box transcription factor 2	HGNC:11597	17q23.2
TBX21	T-box transcription factor 21	HGNC:11599	17q21.32
TBXA2R	thromboxane A2 receptor	HGNC:11608	19p13.3
TCF3	transcription factor 3	HGNC:11633	19p13.3
TCF4	transcription factor 4	HGNC:11634	18q21.2
TCIRG1	T cell immune regulator 1, ATPase H+ transporting V0 subunit a3	HGNC:11647	11q13.2
TCN2	transcobalamin 2	HGNC:11653	22q12.2
TERC	telomerase RNA component	HGNC:11727	3q26.2
TERF2	telomeric repeat binding factor 2	HGNC:11729	16q22.1
TERF2IP	TERF2 interacting protein	HGNC:19246	16q23.1
TERT	telomerase reverse transcriptase	HGNC:11730	5p15.33

TET2	tet methylcytosine dioxygenase 2	HGNC:25941	4q24
TFR2	transferrin receptor 2	HGNC:11762	7q22.1
TFRC	transferrin receptor	HGNC:11763	3q29
TGFB1	transforming growth factor beta 1	HGNC:11766	19q13.2
TGFBR1	transforming growth factor beta receptor 1	HGNC:11772	9q22.33
TGFBR2	transforming growth factor beta receptor 2	HGNC:11773	3p24.1
THBD	thrombomodulin	HGNC:11784	20p11.21
THPO	thrombopoietin	HGNC:11795	3q27.1
THRA	thyroid hormone receptor alpha	HGNC:11796	17q21.1
THRB	thyroid hormone receptor beta	HGNC:11799	3p24.2
TICAM1	TIR domain containing adaptor molecule 1	HGNC:18348	19p13.3
TIMM50	translocase of inner mitochondrial membrane 50	HGNC:23656	19q13.2
TIMM8A	translocase of inner mitochondrial membrane 8A	HGNC:11817	Xq22.1
TINF2	TERF1 interacting nuclear factor 2	HGNC:11824	14q12
TIRAP	TIR domain containing adaptor protein	HGNC:17192	11q24.2
TLN1	talin 1	HGNC:11845	9p13.3
TLR3	toll like receptor 3	HGNC:11849	4q35.1
TLR7	toll like receptor 7	HGNC:15631	Xp22.2
TLR8	toll like receptor 8	HGNC:15632	Xp22.2
TMC6	transmembrane channel like 6	HGNC:18021	17q25.3
TMC8	transmembrane channel like 8	HGNC:20474	17q25.3
TMEFF1	transmembrane protein with EGF like and two follistatin like domains 1	HGNC:11866	9q31.1
TMPRSS15	transmembrane serine protease 15	HGNC:9490	21q21.1
TNFAIP3	TNF alpha induced protein 3	HGNC:11896	6q23.3
TNFRSF11A	TNF receptor superfamily member 11a	HGNC:11908	18q21.33
TNFRSF13B	TNF receptor superfamily member 13B	HGNC:18153	17p11.2
TNFRSF13C	TNF receptor superfamily member 13C	HGNC:17755	22q13.2
TNFRSF1A	TNF receptor superfamily member 1A	HGNC:11916	12p13.31
TNFRSF1B	TNF receptor superfamily member 1B	HGNC:11917	1p36.22
TNFRSF4	TNF receptor superfamily member 4	HGNC:11918	1p36.33
TNFRSF6B	TNF receptor superfamily member 6b	HGNC:11921	20q13.33
TNFRSF9	TNF receptor superfamily member 9	HGNC:11924	1p36.23
TNFSF11	TNF superfamily member 11	HGNC:11926	13q14.11
TNFSF12	TNF superfamily member 12	HGNC:11927	17p13.1
TNFSF13	TNF superfamily member 13	HGNC:11928	17p13.1
TNFSF15	TNF superfamily member 15	HGNC:11931	9q32
TNFSF4	TNF superfamily member 4	HGNC:11934	1q25.1
TNFSF9	TNF superfamily member 9	HGNC:11939	19p13.3
TNIP1	TNFAIP3 interacting protein 1	HGNC:16903	5q33.1
TNPO3	transportin 3	HGNC:17103	7q32.1
TOM1	target of myb1 membrane trafficking protein	HGNC:11982	22q12.3
TONSL	tonsoku like, DNA repair protein	HGNC:7801	8q24.3
TOP2B	DNA topoisomerase II beta	HGNC:11990	3p24.2
TP53	tumor protein p53	HGNC:11998	17p13.1
TP63	tumor protein p63	HGNC:15979	3q28
TPP1	tripeptidyl peptidase 1	HGNC:2073	11p15.4
TPP2	tripeptidyl peptidase 2	HGNC:12016	13q33.1
TRAC	T cell receptor alpha constant	HGNC:12029	14q11.2

TRADD	TNFRSF1A associated via death domain	HGNC:12030	16q22.1
TRAF3	TNF receptor associated factor 3	HGNC:12033	14q32.32
TRAF3IP2	TRAF3 interacting protein 2	HGNC:1343	6q21
TRAPPC2	trafficking protein particle complex subunit 2	HGNC:23068	Xp22.2
TREX1	three prime repair exonuclease 1	HGNC:12269	3p21.31
TRHR	thyrotropin releasing hormone receptor	HGNC:12299	8q23.1
TRIM22	tripartite motif containing 22	HGNC:16379	11p15.4
TRMT10A	tRNA methyltransferase 10A	HGNC:28403	4q23
TRMU	tRNA mitochondrial 2-thiouridylase	HGNC:25481	22q13.31
TRNT1	tRNA nucleotidyl transferase 1	HGNC:17341	3p26.2
TSHB	thyroid stimulating hormone subunit beta	HGNC:12372	1p13.2
TSHR	thyroid stimulating hormone receptor	HGNC:12373	14q24-q31
TSPAN14	tetraspanin 14	HGNC:23303	10q23.1
TSPEAR	thrombospondin type laminin G domain and EAR repeats	HGNC:1268	21q22.3
TSR2	TSR2 ribosome maturation factor	HGNC:25455	Xp11.22
TTC37	SKI3 subunit of superkiller complex	HGNC:23639	5q15
TTC7A	tetratricopeptide repeat domain 7A	HGNC:19750	2p21
TTR	transthyretin	HGNC:12405	18q12.1
TUBB1	tubulin beta 1 class VI	HGNC:16257	20q13.32
TUBGCP3	tubulin gamma complex component 3	HGNC:18598	13q34
TXNRD2	thioredoxin reductase 2	HGNC:18155	22q11.21
TYK2	tyrosine kinase 2	HGNC:12440	19p13.2
TYMS	thymidylate synthetase	HGNC:12441	18p11.32
UBA1	ubiquitin like modifier activating enzyme 1	HGNC:12469	Xp11.3
UBE2L3	ubiquitin conjugating enzyme E2 L3	HGNC:12488	22q11.21
UBE2T	ubiquitin conjugating enzyme E2 T	HGNC:25009	1q32.1
UFD1	ubiquitin recognition factor in ER associated degradation 1	HGNC:12520	22q11.21
UNC119	unc-119 lipid binding chaperone	HGNC:12565	17q11.2
UNC13D	unc-13 homolog D	HGNC:23147	17q25.3
UNC45A	unc-45 myosin chaperone A	HGNC:30594	15q26.1
UNC93B1	unc-93B1 regulator of TLR signaling	HGNC:13481	11q13.2
UNG	uracil DNA glycosylase	HGNC:12572	12q24.11
USB1	U6 snRNA biogenesis phosphodiesterase 1	HGNC:25792	16q21
USF3	upstream transcription factor family member 3	HGNC:30494	3q13.2
USP18	ubiquitin specific peptidase 18	HGNC:12616	22q11.21
USP43	ubiquitin specific peptidase 43	HGNC:20072	17p13.1
USP48	ubiquitin specific peptidase 48	HGNC:18533	1p36.12
USP8	ubiquitin specific peptidase 8	HGNC:12631	15q21.2
VAMP1	vesicle associated membrane protein 1	HGNC:12642	12p13.31
VAV1	vav guanine nucleotide exchange factor 1	HGNC:12657	19p13.3
VIPAS39	VPS33B interacting protein, apical-basolateral polarity regulator, spe-39 homolog	HGNC:20347	14q24.3
VPS13B	vacuolar protein sorting 13 homolog B	HGNC:2183	8q22.2
VPS33A	VPS33A core subunit of CORVET and HOPS complexes	HGNC:18179	12q24.31
VPS33B	VPS33B late endosome and lysosome associated	HGNC:12712	15q26.1
VPS45	vacuolar protein sorting 45 homolog	HGNC:14579	1q21.2
VSIG4	V-set and immunoglobulin domain containing 4	HGNC:17032	Xq12
VTN	vitronectin	HGNC:12724	17q11.2
WAS	WASP actin nucleation promoting factor	HGNC:12731	Xp11.23

WDR1	WD repeat domain 1	HGNC:12754	4p16.1
WDR44	WD repeat domain 44	HGNC:30512	Xq24
WIPF1	WAS/WASL interacting protein family member 1	HGNC:12736	2q31.1
WNT2B	Wnt family member 2B	HGNC:12781	1p13.2
WNT6	Wnt family member 6	HGNC:12785	2q35
WRAP53	WD repeat containing antisense to TP53	HGNC:25522	17p13.1
WWTR1	WW domain containing transcription regulator 1	HGNC:24042	3q25.1
XIAP	X-linked inhibitor of apoptosis	HGNC:592	Xq25
XRCC2	X-ray repair cross complementing 2	HGNC:12829	7q36.1
XRCC4	X-ray repair cross complementing 4	HGNC:12831	5q14.2
YARS2	tyrosyl-tRNA synthetase 2	HGNC:24249	12p11.21
ZAP70	zeta chain of T cell receptor associated protein kinase 70	HGNC:12858	2q11.2
ZBTB24	zinc finger and BTB domain containing 24	HGNC:21143	6q21
ZC3HC1	zinc finger C3HC-type containing 1	HGNC:29913	7q32.2
ZCCHC8	zinc finger CCHC-type containing 8	HGNC:25265	12q24.31
ZEB2	zinc finger E-box binding homeobox 2	HGNC:14881	2q22.3
ZFP36	ZFP36 zinc finger CCCH-type	HGNC:12862	19q13.2
ZFP57	ZFP57 zinc finger protein	HGNC:18791	6p22.1
ZMYND10	zinc finger MYND-type containing 10	HGNC:19412	3p21.31
ZNF34	zinc finger protein 34	HGNC:13098	8q24.3
ZNF341	zinc finger protein 341	HGNC:15992	20q11.22
ZNF699	zinc finger protein 699	HGNC:24750	19p13.2
ZNFX1	zinc finger NFX1-type containing 1	HGNC:29271	20q13.13

Supplementary Table S5. Urinalysis.

**Parameter, units**	**2023**	**November 2024**	**May 2025**	**Reference range**
Color	–	–	Light yellow	Light yellow – yellow
Clarity	–	–	Clear	Clear
Specific gravity	–	–	1.007	1.010–1.025
Reaction (pH)	–	–	7.0 (neutral)	5.0–7.0 (slightly acidic – neutral)
Protein, g/L	–	1.61	4.3	<0.15
Glucose, mmol/L	–	–	Normal	Normal (<2.8)
Ketones, mmol/L	–	–	Negative	Negative
Occult blood	–	–	Negative	Negative
Bilirubin	–	–	Negative	Negative

Urobilinogen	–	–	Normal	Normal (<34)
Nitrites	–	–	Negative	Negative
Leukocytes	–	–	Negative	Negative
Squamous epithelium, cells/HPF	–	–	3	0–5
Leukocytes, cells/HPF	–	–	2	0–5
Erythrocytes, cells/HPF	3	3	2	0–1

Note: HPF – High-Power Field.
